# Visualizing Influenza A Virus vRNA Replication

**DOI:** 10.3389/fmicb.2022.812711

**Published:** 2022-06-06

**Authors:** Ya-Fang Chiu, Yi-Wen Huang, Chi-Yuan Chen, Yu-Chia Chen, Yu-Nong Gong, Rei-Lin Kuo, Chung-Guei Huang, Shin-Ru Shih

**Affiliations:** ^1^Department of Microbiology and Immunology, Chang Gung University, Taoyuan, Taiwan; ^2^Research Center for Emerging Viral Infections, Chang Gung University, Taoyuan, Taiwan; ^3^Department of Laboratory Medicine, Linkou Chang Gung Memorial Hospital, Taoyuan, Taiwan; ^4^Department of Biochemical Science and Technology, College of Life Science, National Taiwan University, Taipei, Taiwan

**Keywords:** apoptosis, influenza A virus, live-cell imaging, viral-host interaction, vRNA

## Abstract

Influenza A virus (IAV) has caused recurrent epidemics and severe pandemics. In this study, we adapted an MS2-MCP live-cell imaging system to visualize IAV replication. A reporter plasmid, pHH-PB2-vMSL, was constructed by replacing a part of the PB2-coding sequence in pHH-PB2 with a sequence encoding 24 copies of a stem-loop structure from bacteriophage MS2 (MSL). Binding of MS2 coat protein (MCP) fused to green fluorescent protein (GFP) to MSL enabled the detection of vRNA as fluorescent punctate signals in live-cell imaging. The introduction of pHH-PB2-vMSL into A549 cells transduced to express an MCP-GFP fusion protein lacking the nuclear localization signal (MCP-GFPdN), subsequently allowed tracking of the distribution and replication of PB2-vMSL vRNA after IAV PR8 infection. Spatial and temporal measurements revealed exponential increases in vRNA punctate signal intensity, which was only observed after membrane blebbing in apoptotic cells. Similar signal intensity increases in apoptotic cells were also observed after MDCK cells, transduced to express MCP-GFPdN, were infected with IAV carrying PB2-vMSL vRNA. Notably, PB2-vMSL vRNA replication was observed to occur only in apoptotic cells, at a consistent time after apoptosis initiation. There was a lack of observable PB2-vMSL vRNA replication in non-apoptotic cells, and vRNA replication was suppressed in the presence of apoptosis inhibitors. These findings point to an important role for apoptosis in IAV vRNA replication. The utility of the MS2-imaging system for visualizing time-sensitive processes such as viral replication in live host cells is also demonstrated in this study.

## Introduction

Influenza A virus (IAV) is a major human pathogen of fascinating complexity. This virus belongs to the *Orthomyxoviridae* family and contains a lipid envelope acquired from the host cell plasma membrane during budding that surrounds a layer of matrix 1 (M1) protein (Palese, 1977; Palese and Shaw, 2007), inside of which are the core viral ribonucleoprotein (vRNP) complexes, consisting of eight negative-sense viral RNAs (vRNA) wrapped in viral nucleoprotein (NP). Both termini of each vRNA are bound together by the heterotrimeric viral RNA-dependent RNA polymerase (RdRP), which is composed of PB1, PB2, and PA viral polymerase subunits (Palese, 1977; Palese and Shaw, 2007). Each vRNA encodes at least one viral protein that is essential for IAV production (Palese, 1977; Palese and Shaw, 2007); among these proteins, hemagglutinin (HA) is involved in receptor binding and membrane fusion, neuraminidase (NA) is required for viral release, matrix-2 (M2) is an ion-channel protein, non-structural protein 1 (NS1) acts against cellular antiviral responses, and matrix-1 (M1) and non-structural protein 2 (NS2) are involved in the nuclear export of vRNP (Lamb and Lai, 1980; Lamb et al., 1981). These components are key to the propagation of IAV and play critical roles in the IAV infectious cycle; however, the processes they are involved in are often time-sensitive, and are thus difficult to track using conventional methods. Therefore, we sought to establish a live-cell imaging approach that could better elucidate each step in the IAV infectious cycle.

Different modes of live-cell imaging have been used in the past to shed light on important facets of the IAV life cycle, including the dynamic processes of infection and entry, import of vRNAs from the cytoplasm to the nucleus for transcription and replication, and trafficking and assembly (Lakadamyali et al., 2003; Chen and Zhuang, 2008; Li et al., 2010). For instance, IAV trafficking after infection has been imaged through the use of a lipophilic dye or a tetracysteine peptide, which were, respectively, incorporated into the viral membrane or the NS1 and NP proteins (Lakadamyali et al., 2003; Chen and Zhuang, 2008; Li et al., 2010; Han et al., 2021). However, this method is limited by the short half-life of fluorophores, as well as interference with viral genome assembly from the incorporated peptide sequences (Lakadamyali et al., 2003; Chen and Zhuang, 2008; Li et al., 2010; Han et al., 2021). Moreover, the incorporation of tetracysteine influences the distribution of NS1, which may affect viral trafficking processes (Li et al., 2010). Several attenuated IAV strains, with either NS1 or PA fused with an eGFP reporter, have been generated to, respectively, investigate tropism and assembly processes (Lakdawala et al., 2014; De Baets et al., 2015). A recent study has also shown that individual segments of the IAV genome can be detected using single-molecule fluorescence *in situ* hybridization (smFISH), an approach that has led to a better understanding of the intracellular trafficking mechanisms that mediate the interactions between vRNAs during packaging and reassortment (Chou et al., 2012). However, better approaches are still needed to investigate the kinetics of IAV-host cell interactions.

Live-cell imaging is a method that uses time-lapse microscopy to study the dynamics of biological events in signal live cells using microscopes equipped with a light source that does not damage cells. Unlike conventional biochemical analysis, which studies a phenomenon in a population of cells and takes an average of the results, live-cell imaging follows signal cells in real time and reveals information that cannot be provided by conventional methods. In this study, we adapted an approach that was successfully used to image the HIV genome, and sought to apply it to the investigation of IAV vRNA replication. The original approach incorporated 24 copies of an RNA stem-loop structure (MSL) derived from the MS2 RNA bacteriophage into the HIV genome (Chen et al., 2009, 2014, 2016; Sardo et al., 2015; Becker and Sherer, 2017; Behrens et al., 2017). The binding of the MS2 coat protein (MCP) to MSL, which contains a ribosomal-binding site, inhibits the translation of the downstream replicase gene in MS2 (Weissmann et al., 1963; Fiers et al., 1976; Campbell and Patel, 1983; Uhlenbeck et al., 1983). Fusion of green fluorescent protein (GFP) with the MCP enables the florescent detection of HIV RNAs in single live cells following MCP binding to MSL (Chen et al., 2009, 2014, 2016; Sardo et al., 2015; Gardiner et al., 2016; Pocock et al., 2016; Becker and Sherer, 2017; Behrens et al., 2017). In our adapted parallel approach, MSL was incorporated into IAV vRNA to allow visualization of vRNA replication. This approach offers an advantage over previous methods in that GFP has a considerably longer half-life than fluorescent dyes (Corish and Tyler-Smith, 1999), while the incorporation of MSL has minimal effects on viral genome assembly (Chen et al., 2020).

IAV is known to activate apoptosis in host cells after infection (Schultz-Cherry and Hinshaw, 1996; Morris et al., 1999; Schultz-Cherry et al., 2001; Zamarin et al., 2005; Halder et al., 2011; Muramoto et al., 2013; Gao et al., 2015; Nailwal et al., 2015; Nacken et al., 2017; Pasricha et al., 2018), and earlier studies have shown that apoptosis activation can be attributed to the high-level expression of NS1, NA, and PB1-F2 (Schultz-Cherry and Hinshaw, 1996; Morris et al., 1999; Schultz-Cherry et al., 2001; Zamarin et al., 2005; Muramoto et al., 2013; Nacken et al., 2017; Pasricha et al., 2018). In addition, M1 is known to prevent interaction between the 70-kilodalton (kDa) heat shock protein (HSP70) and APAF-1 to induce the formation of the apoptosome (Halder et al., 2011), while IAV-encoded PA-X enhances inflammatory responses to promote apoptosis (Gao et al., 2015), and NP can repress expression of the RNF43 ubiquitin E3 ligase to induce p53 signaling and apoptosis (Nailwal et al., 2015). Interestingly, unlike the other IAV proteins, NS1 has dual functions, and can either prevent or induce apoptosis after coordinating with other host and viral factors (Schultz-Cherry et al., 2001; Zhirnov et al., 2002; Jackson et al., 2010; Nacken et al., 2017).

The impact of apoptosis on IAV replication and propagation in host cells is not well defined as yet (Atkin-Smith et al., 2018). It is well-established that cells often use apoptosis to limit replication of viruses (Barber, 2001; Kerr et al., 2002; Chattopadhyay et al., 2011, 2016), and the prevention of apoptosis by NS1 (Zhirnov et al., 2002; Jackson et al., 2010), therefore, apoptosis can serve as a host defense against IAV proliferation. However, under some circumstances, the induction of apoptosis may serve to facilitate viral replication (Atkin-Smith et al., 2018; Ning et al., 2019), and many studies indicate that apoptosis may actually benefit IAV propagation. A previous study found that IAV infection induces both autophagy and Bax/Bak-dependent apoptosis in A549 cells, and the autophagy pathway appeared to be critical to IAV production; inhibition of the pathway subsequently blocked both IAV-induced apoptosis and IAV vRNA propagation (Yeganeh et al., 2018). Other studies have shown that inhibition of IAV-induced apoptosis impaired viral production (Wurzer et al., 2003, 2004; McLean et al., 2009; Tran et al., 2020). It is likely that different cell death parameters used to evaluate apoptosis, as well as the different pathways (Laghlali et al., 2020) and stages of apoptosis observed, may have contributed to the conflicting evidence currently available, and thus a live-cell imaging approach can help to provide time-specific results that cannot be assessed using biochemical studies.

## Materials and Methods

### Cell Lines

Human embryonic kidney 293T (HEK293T), Madin-Darby Canine Kidney (MDCK), and A549 adenocarcinomic human alveolar basal epithelial cells, as well as derivative MDCK(PB2) and A549(MCP-GFPdN) cells, were cultured in Dulbecco’s modified Eagle medium (DMEM) supplemented with 10% fetal bovine serum (Hyclone).

### Plasmids

Plasmid pCR4-24XMS2SL-stable was provided by Robert Singer (Addgene plasmid #31865; Bertrand et al., 1998). Plasmid pHH-PB2 contains the PB2 cDNA transcribed from an RNA polymerase I (Pol I) promoter and was constructed according to the method described elsewhere (Neumann et al., 1999). A Klenow-filled, *Bam*HI-*Bgl*II DNA fragment encoding 24 copies of MSL from pCR4-24xMS2SL-stable was inserted into the *Pvu*II-*Bgl*II sites of pHH-PB2 to generate pHH-PB2-vMSL. MSL was similarly inserted but in a reverse orientation to generate pHH-PB2-mMSL. The PB2 gene was amplified by PCR with primers 5′-GCACGGCCGAGCGAAAGCAGGTCAATTA and 5′-ATAGGATCCAGTA GAAACAAGGTCGTTT, using pDZ-PB2 as a template. The fragment was digested with *Eag*I and *Bam*HI and inserted into the *Not*I-*Bam*HI sites in pQCXIH (Clontech) to yield pQCXIH-PB2, to enable transcription of PB2 mRNA from the cytomegalovirus (CMV) immediate-early promoter. Plasmid pMCP-GFP was kindly provided by Wei-Shau Hu (National Cancer Institute, United States). The DNA sequence encoding the nuclear localization signal (NLS) was deleted from MCP-GFP and cloned into the *Hpa*I-*Not*I sites of a retroviral vector, p3048 (kindly provided by Bill Sugden), to generate pMCP-GFPdN. Plasmids pDZ-PA, pDZ-PB1, pDZ-NP, pDZ-HA, pDZ-NA, pDZ-M, and pDZ-NS were described elsewhere (Quinlivan et al., 2005).

### Generating A549(MCP-GFPdN) and MDCK(PB2) Cells

Retrovirus carrying the MCP-GFPdN gene was generated in HEK293T cells as previously described (Vereide et al., 2014). HEK293T cells (5 × 10^6^) were cotransfected with 10 μg of pMCP-GFPdN, 1 μg of p1238, which expresses p50/p65, 1 μg of p2842, a VSV-g expression plasmid, and 3 μg of p2843, which expresses gag-pol (Vereide et al., 2014), using DharmaFECT kb DNA transfection reagent (Dharmacon). Retroviruses were collected 3 days post-transfection and stored at −80°C. A549 and MDCK cells were infected by retroviruses encoding MCP-GFPdN, and then sorted with a cell sorter (FACSAriaIIu, Becton Dickinson) according to the intensity of green fluorescence; the 30% of cells with low green fluorescence was selected to generate the A549(MCP-GFPdN) and MDCK(MCP-GFPdN) cell lines, respectively. MDCK(MCP-GFPdN) cells were infected with retroviruses encoding PB2 and selected with 200 µg/ml of Hygromycin to generate MDCK(PB2) cells.

### IAV Production From HEK293T

HEK293T cells (2 × 10^6^) were cocultured with 6 × 10^5^ MDCK(PB2) cells in a 6-cm cell culture dish. Cells were cotransfected using DharmaFECT kb DNA transfection reagent with 1 μg each of pQCXIH-PB2 (encoding PB2), pDZ-PB1, pDZ-PA, pDZ-HA, pDZ-NP, pDZ-NA, pDZ-M, and pDZ-NS along with pHH21 (an empty vector), pHH-PB2, or pHH-PB2-vMSL, respectively, to produce the IAV derivatives, IAV(null), IAV(PB2), or IAV(PB2-vMSL). At 24 h post-transfection, cells were washed twice with serum-free DMEM, and were then maintained in serum-free DMEM containing 1 μg/ml tosylsulfonyl phenylalanyl chloromethyl ketone (TPCK)-treated trypsin for 3 days.

### Fluorescence Microscopy

HEK293T cells were grown on coverslips and cotransfected with plasmids. At 16 h post-transfection, cells were fixed with 4% paraformaldehyde in phosphate-buffered saline (PBS) for 20 min at room temperature, embedded in CitiFluor AF1 (Agar Scientific), and observed under a Nikon TiE eclipse inverted microscope using a 100× Apo TIRF oil objective (NA = 1.49) lens. Images were captured using an ORCA-Flash 4.0 Digital CMOS camera-C11440-22CU (Hamamatsu). Images were acquired under 25% LED light with 400 ms exposure. Fluorescence signals in z-planes were captured and analyzed.

### Time-Lapse Experiments

To monitor PB2-vMSL vRNA replication in live cells, 8 × 10^4^ A549(MCP-GFPdN) cells were seeded in an Attofluor cell chamber (Invitrogen), cultured overnight, and transfected with pHH-PB2-vMSL. At 20 h post-transfection, cells were washed twice with serum-free DMEM, infected with PR8 strain of IAV at a multiplicity of infection (MOI) of 2 in the presence of TPCK-trypsin, cultured in the Tokai Hit stage top incubation system (TOKAI HIT Co. Ltd.) with precision temperature, humidity and CO_2_ control, and imaged in a time-lapse experiment using a Nikon Ti eclipse inverted microscope. During the imaging process, cells were maintained in serum-free DMEM with 200 ng/ml of TPCK-trypsin. Forty z-planes separated by 0.3 μm were acquired every 60 min for 18 h. For visualizing vRNA replication in MDCK(PB2) cells after IAV infection, cells were imaged every 30 min for 48 h. We also added 2 μg/ml of Hoechst 33342 into culture medium to stain live cells in a 37°C incubator for 1 h. After washing twice with medium to remove unbound dye, cells were infected with IAV and subsequently imaged. Images were acquired under 25% LED light with 300 ms of exposure time for green signals, so as to facilitate the detection of signals with minimal exposures. Images were taken with an Apo TRIF 100×, 1.49 NA oil objective, and an ORCA-Flash 4.0 Digital CMOS camera-C11440-22CU. No image was saturated, as judged by their most intense pixels being less than half of approximately 65,000 gray levels. Images were displayed as square of maximum intensity projections across multiple z-planes in ImageJ (Schneider et al., 2012). All images acquired in live cells were analyzed with the Computer-Assisted Plasmid Summation (CAPS) program to measure the intensity of punctate signals, which uses photometry with subtraction of background signals derived from an annulus of the pixels over multiple z-planes surrounding a given signal from PB2-vMSL vRNA (Chiu et al., 2013).

### Quantifying vRNA in Virions by RT-qPCR

vRNA was isolated using the QIAamp Viral RNA kit (Qiagen) according to the manufacturer’s instructions, and reverse transcribed using SuperScript IV reverse transcriptase (Invitrogen) with a specific primer annealing to PB2 vRNA (5′-ATCTAATGTCGCAGTCTCGC). RT-qPCR was performed using a QuantiFast SYBR Green PCR kit (Qiagen) with specific primers for detecting PB2 vRNA.

### RNA Preparation for RNA-Seq

RNA was isolated from A549 cells infected by IAV at an MOI of 2 using TRIzol Reagent according to the manufacturer’s instructions (Invitrogen). The MGIEasy RNA Directional Library Prep Set User Manual (MGI) was used to prepare cDNA libraries. Equal concentrations of each library were sequenced using a DNBSEQ-G400 (MGI) platform to create pair-end 100-bp reads. Sequences were assessed for quality and trimmed for primer-adaptor sequences using an RNA-seq alignment tool from MagaBolt (MGI), followed by alignment to the human reference genome (hg38). Nucleotide sequences of the eight PR8 segments were concatenated to generate a single sequence for read mapping using bowtie2 (version 2.4.4, default setting with very-sensitive option; Langmead and Salzberg, 2012). Samtools (version 1.7; Li et al., 2009) was applied to generate the pileup file and calculate read distribution. NGS coverage and depth were visualized using the R package ggplot2 (Wickham, 2009). The BioProject accession number is PRJNA786297.^1^

### Flow Cytometric Study of vRNA Replication

A549 cells were infected with PR8 at an MOI of 2. Cells were stained with Annexin V Apoptosis Detection Kit FITC according to manufacturer’s procedure (eBioscience) at the indicated time points post-infection. Apoptotic and non-apoptotic cells were sorted with a FACSAria IIu cell sorter (BectonDickinson).

## Results

### Constructing pHH-PB2-vMSL for Visualizing IAV vRNA

**Figure 1:**
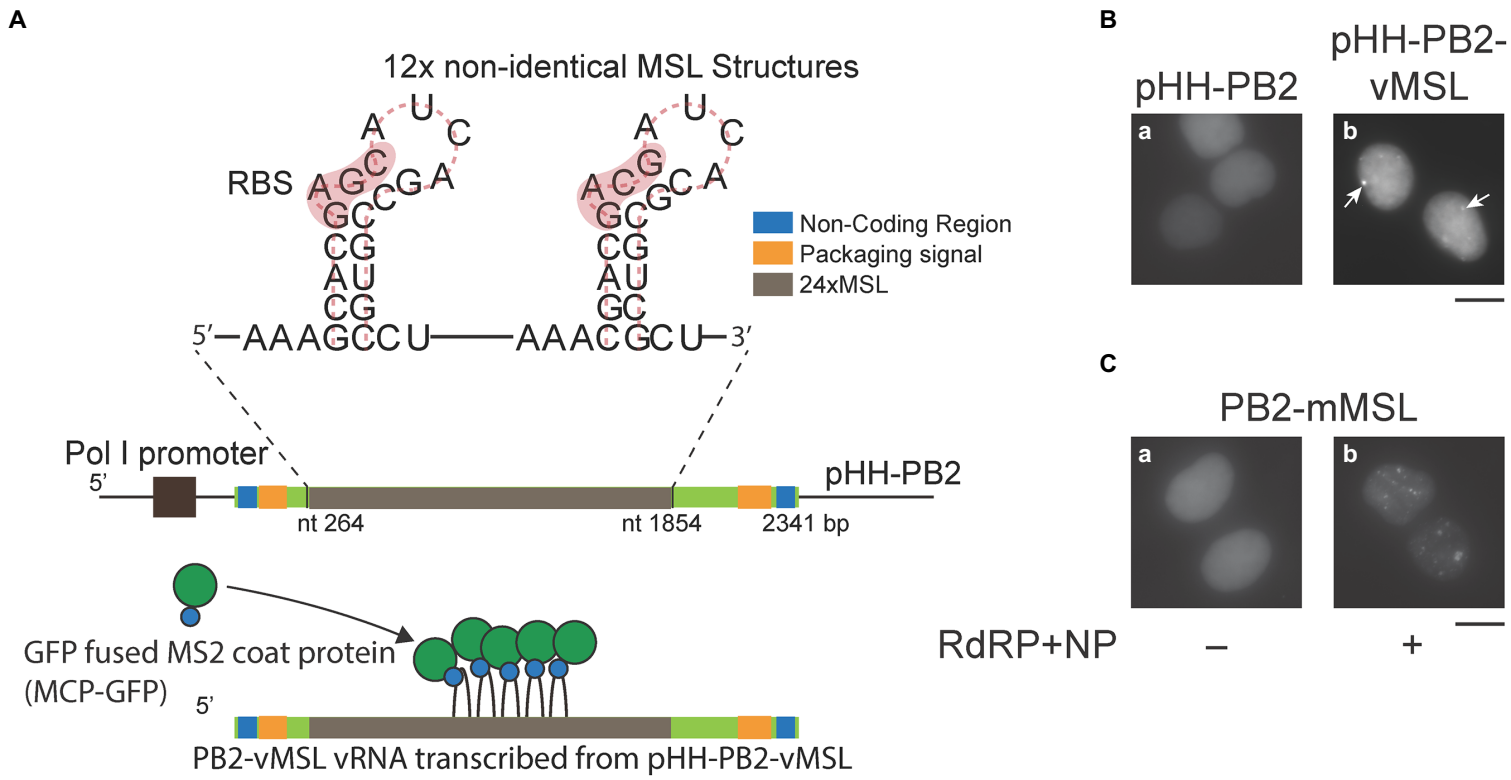
An imaging system for visualizing influenza A virus (IAV) vRNA. **(A)** Twelve copies of a DNA fragment encoding two tandemly located non-identical MSL structures, which contain a ribosomal-binding site (RBS) and the translation initiation codon of the MS2 bacteriophage replicase gene, were used to replace a portion of the PB2 gene in pHH-PB2 to generate pHH-PB2-vMSL. PB2-vMSL vRNA is transcribed from the RNA polymerase I (POL I) promoter, and the MSL structures can be bound by MCP-GFP fusion protein. **(B)** HEK293T cells were cotransfected with pMCP-GFP and **(a)** pHH-PB2 or **(b)** pHH-PB2-vMSL. Cells were fixed with paraformaldehyde and observed at 16 h after transfection. Arrows indicate punctate signals. **(C)** HEK293T cells were cotransfected with pMCP-GFP and **(a)** pHH-PB2-mMSL or **(b)** pHH-PB2-mMSL and plasmids expressing NP and the RdRP components PB1, PB2, and PA. Cells were fixed at 16 h after transfection. The images of representative cells were acquired under a Nikon TiE eclipse inverted microscope. Scale bar: 10 μm.

In order to visualize IAV vRNA in real time, a part of the PB2 cDNA in pHH-PB2 (Neumann et al., 1999), which transcribes PB2 vRNA from an RNA polymerase I (POL I) promoter, was substituted with a DNA fragment encoding 12 copies of two tandemly located non-identical MSL structures (Fusco et al., 2003) to create pHH-PB2-vMSL (Figure 1A). HEK293T cells were subsequently cotransfected with pHH-PB2-vMSL and pMCP-GFP (Fusco et al., 2003), which expresses an MSL-binding protein fused to GFP (MCP-GFP). At 16 h after transfection and fixation, PB2-vMSL vRNA was microscopically visualized as green fluorescent punctate signals (Figure 1Bb). The MCP-GFP used herein was engineered to contain the nuclear localization signal (NLS) (Fusco et al., 2003), and thus only the nucleus exhibits green fluorescence (Figure 1B). We examined about 300 cells in each of the three independent experiment and found that 39%–48% of the cells had signals in the nucleus; none had signals in the cytoplasm (Supplementary Table 1). The percentage of cells with signals increased to 65%–71% with no cell with signals in the cytoplasm if the cells were cotransfected with plasmids encoding RdRP and NP; 55%–79% if the cells were cotransfected with all the IAV plasmids (Supplementary Table 1). In parallel experiments, HEK293T cells were cotransfected with pHH-PB2-vMSL, pMCP-GFPdN, which expresses an MCP-GFP lacking an NLS (MCP-GFPdN), and all IAV plasmids. Interestingly, MCP-GFP seemed to prevent the PB2-vMSL RNP from exiting the nucleus in HEK293T cells unless the NLS is deleted from MCP-GFP (Supplementary Table 1). As expected, fluorescent punctate signals were not visible in cells cotransfected with pMCP-GFP and pHH-PB2 (Figure 1Ba), which lacks the MSL sequence and therefore cannot recruit the MSL-binding MCP-GFP fusion protein. We also cotransfected HEK293T cells with pHH-PB2-vMSL and plasmids expressing NP and RdRP, and the amount of vRNA was determined by RT-qPCR at 24 h after transfection. The results showed that when the PB2 sequence was replaced with MSL, the amount of PB2-vMSL increased by 2.7-fold in the presence of RdRP and NP (Supplementary Figure 1). However, replacement of the sequence appeared to decrease the efficiency of replication due to the presence of stem-loop structures, as in a similar experiment, the increase in unreplaced PB2 vRNA was 8.3-fold higher than for PB2-vMSL, suggesting that PB2-vMSL is not replicated as efficiently as PB2 vRNA (Supplementary Figure 1).

After infection, IAV is known to facilitate vRNA replication by transcribing positive-sense mRNA and cRNA, using vRNA as a template. To ascertain that MCP-GFP binds to only PB2-vMSL vRNA but not PB2-vMSL mRNA and cRNA, we inserted a DNA fragment encoding the sequence complementary to MSL into pHH-PB2, to derive pHH-PB2-mMSL. Following cotransfection of HEK293T cells with pHH-PB2-mMSL and pMCP-GFP, punctate signals remained undetected at 16 h post-transfection (Figure 1Ca), thereby demonstrating that MCP-GFP does not bind to vRNA containing MSL complementary sequences. We further cotransfected HEK293T cells with pHH-PB2-mMSL, pMCP-GFP, and plasmids expressing NP, and the RdRP components (PB1, PB2, and PA), in order to facilitate the transcription of PB2-mMSL mRNA and cRNA, which contained MSL instead of its complementary sequence. At 16 h post-transfection, green fluorescent punctate signals were detected in the cells (Figure 1Cb), indicative of MCP-GFP binding to MSL structures on PB2-mMSL mRNA and cRNA. These results show that MCP-GFP binds solely to MSL structures in PB2-vMSL vRNA, but not those formed by MSL complementary sequences in PB2-vMSL mRNA and cRNA, and therefore this live-cell imaging system can be confirmed to detect only PB2-vMSL vRNA.

**Figure 2:**
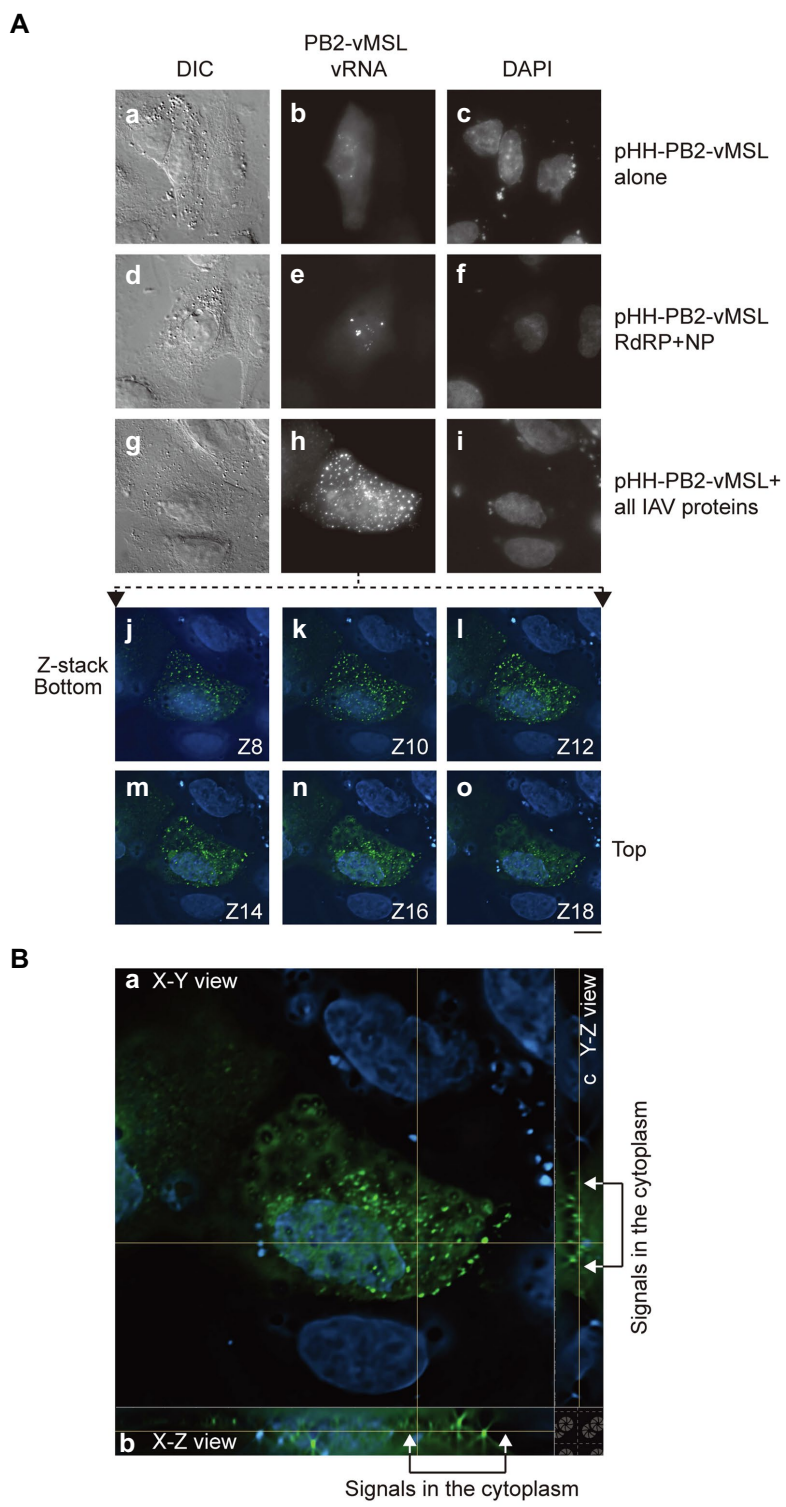
Distribution of PB2-vMSL vRNA punctate signals in A549 cells. **(A)** A549 cells were co-transfected with **(a–c)** pHH-PB2-vMSL and pMCP-GFPdN; **(d–f)** pHH-PB2-vMSL, pMCP-GFPdN, and plasmids expressing NP and the RdRP components, PB1, PB2, and PA; or **(g–i)** pHH-PB2-vMSL, pMCP-GFPdN, and eight plasmids encoding all IAV proteins (pQCXIH-PB2, pDZ-PB1, pDZ-PA, pDZ-NP, pDZ-HA, pDZ-NA, pDZ-M, and pDZ-NS). The images of representative cells are shown as the square of maximum intensity projections across the z-planes in ImageJ. The signals of the representative cell **(g–i)** in multiple Z planes were shown **(j–o)**. **(B)** The signals in the representative cell **(g–i)** were deconvoluted with NIS-elements v5.2 (Nikon) and shown in orthogonal sectioning of a z-stack: **(a)** XY view; **(b)** XZ view; **(c)** YZ view. Cells were fixed with paraformaldehyde at 16 h post-transfection. The nucleus was stained with DAPI. Differential interference contrast (DIC) and fluorescence images were acquired using a Nikon TiE eclipse inverted microscope, using the same settings as those employed for Figure 1. Scale bar: 10 μm.

In control experiments, cotransfecting HEK293T cells with individual reverse genetics and pHH-PB2-vMSL or pHH-PB2-mMSL did not yield punctate signals (Supplementary Figure 2). Only those cells transfected with pHH-PB2-vMSL (Supplementary Figure 2Ak; Figure 2Bk), or cotransfected with NP, PB1, PB2, and PA plasmids had signals (Supplementary Figures 2Al, 2Bl,Bp), showing that transfecting the cells with individual plasmids does not produce background signals.

### Distribution of PB2-vMSL vRNA

To ascertain whether PB2-vMSL vRNA is similarly exported from the nucleus as parental IAV PB2 vRNA, A549 cells were cotransfected with pHH-PB2-vMSL and pMCP-GFPdN, and at 16 h post-transfection, cells were fixed with paraformaldehyde and examined microscopically, using the same microscope settings as those employed for Figure 1. We observed that 65%–80% of GFP-positive cells had punctate signals, and that cotransfection of A549 cells with pHH-PB2-vMSL and pMCP-GFPdN did not result in the nuclear export of vRNA (Figure 2a–c; Supplementary Table 1). Note that PB2-vMSL vRNA can localize in the nucleus even in the absence of viral proteins, and these punctate signals are formed by the recruitment and binding of MCP-GFPdN to the MSL in the nucleus. Even in cells additionally cotransfected with the RdRP and NP plasmids (pDZ-PA, pDZ-PB1, pQCXIH-PB2, and pDZ-NP), all punctate signals were confined to the nucleus (Figure 2d–f; Supplementary Table 1), although signal intensity did increase (Figure 2e). Earlier studies have shown that vRNA nuclear export requires M1 and NS2 (Lamb and Lai, 1980; Lamb et al., 1981), and thus cotransfection of plasmids expressing NP and RdRP components alone are insufficient to support the nuclear export of PB2-vMSL vRNA. A follow-up experiment in which pHH-PB2-vMSL and pMCP-GFPdN were cotransfected with eight plasmids expressing all IAV proteins (pDZ-PA, pDZ-PB1, pQCXIH-PB2, pDZ-NP, pDZ-HA, pDZ-NA, pDZ-M, and pDZ-NS) demonstrated the presence of punctate signals (7%–18%) in the cytoplasm (Figure 2h; Supplementary Table 1). Imaging in different z-planes (Figure 2j–o) and in orthogonal sectioning (the XZ and YZ images constructed to correspond to an area of interest in an XY image following collection of a z stack of images; Figure 2B) also showed that these dots were present outside of the nucleus, verifying the presence of PB2-vMSL vRNA in the cytoplasm (Figure 2h; Supplementary Table 1). These results indicated the nuclear export of PB2-vMSL vRNA in the presence of all IAV proteins.

We further transduced A549 cells to constitutively express MCP-GFPdN, thereby establishing A549(MCP-GFPdN) cells. These cells were transfected with pHH21 or pHH-PB2-vMSL, followed by infection with two MOI of PR8 IAV at 20 h post-transfection. At 6 h after infection, cells were fixed with paraformaldehyde and immunostained for NP. The results showed that the presence of pHH-PB2-vMSL did not change the distribution pattern of NP, which was present in the entire cell but enriched in the nucleus (Supplementary Figure 3).

### Apoptosis Induced by IAV Infection

**Figure 3:**
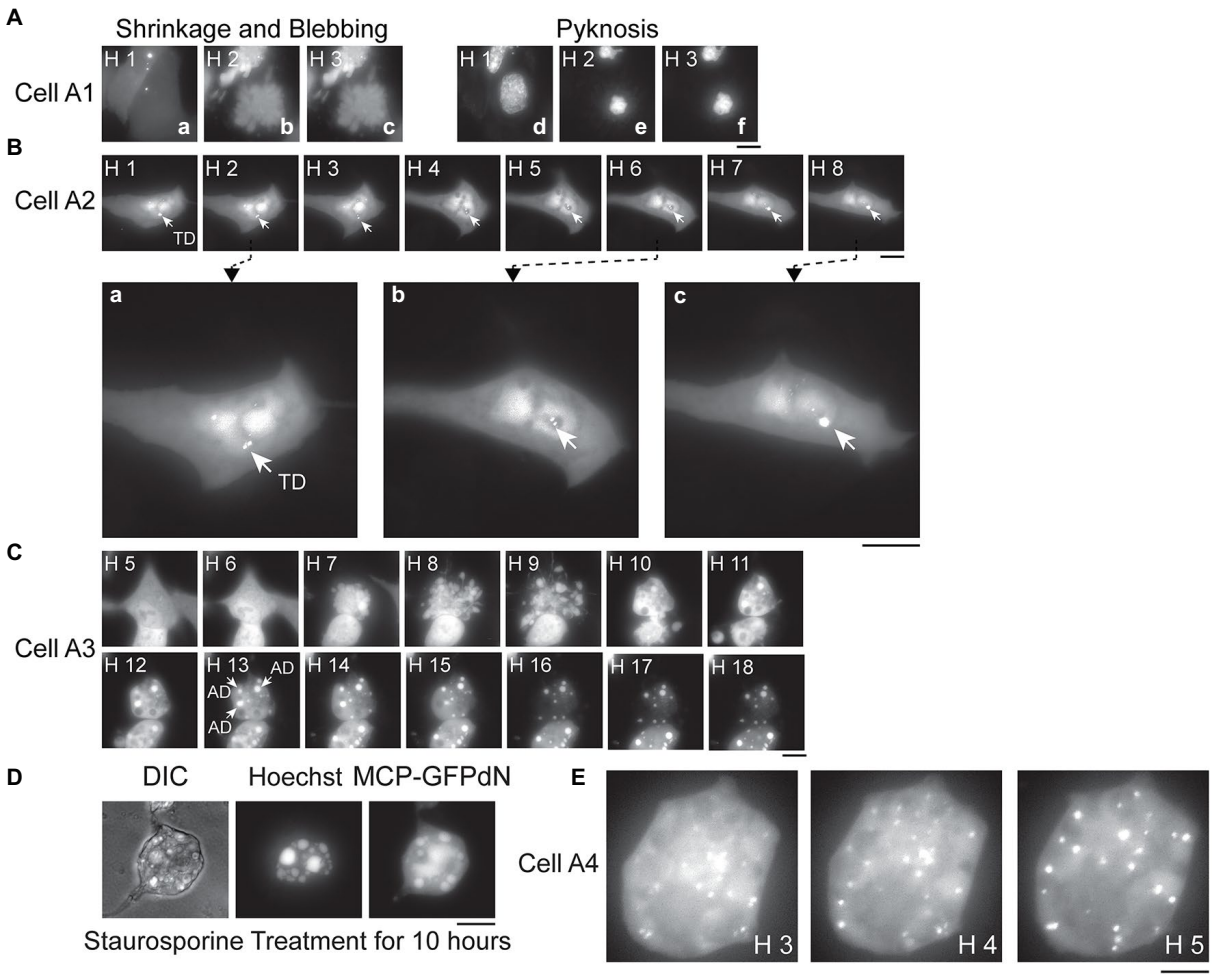
Apoptosis and signals derived from transcribed or replicated PB2-vMSL vRNA. **(A)** A549(MCP-GFPdN) cells were infected with IAV, and the images of representative Cell A1 were taken from Hour 1 to 3 after IAV infection. Images **a–c** show the fluorescent signals displayed by Cell A1, and which reveal cell shrinkage and blebbing. Images **d–f** show pyknosis of the nucleus stained with Hoechst 33342. **(B)** A549(MCP-GFPdN) cells were transfected with pHH-PB2-vMSL, and at 16 h after transfection, cells were imaged every hour for 8 h, with the images derived from representative Cell A2 shown here. The three images, respectively, taken at **(a)** Hour 2, **(b)** Hour 6, and **(c)** Hour 8 post-infection have been enlarged to facilitate viewing, with arrows indicating transcription dots (TDs) generated by PB2-vMSL vRNA transcribed from pHH-PB2-vMSL. **(C)** A549(MCP-GFPdN) cells were infected by IAV, and the images of apoptotic dots (ADs; indicated by arrows) from representative Cell A3 were taken hourly for 18 h. Note that not all ADs in the cell are indicated. **(D)** A549(MCP-GFPdN) cells were treated with staurosporine for 10 h to induce apoptosis, and stained with Hoechst 33342 for 30 min before paraformaldehyde fixation. **(E)** A549(MCP-GFPdN) cells were transfected with pHH-PB2-vMSL, then infected with IAV at 20 h post-transfection and subsequently imaged. Representative Cell A4 displayed blebbing of the plasma membrane at Hour 2 post-infection, and almost all fluorescent signals in the representative images shown here consist of replicated vRNA dots (RDs). Images were acquired using a Nikon TiE eclipse inverted microscope, and images at the indicated time points are displayed as the square of maximum intensity projections across the z-planes in ImageJ. Scale bar: 10 μm.

It is known that IAV infection induces apoptosis in host cells (Barber, 2001; Kerr et al., 2002; Chattopadhyay et al., 2011, 2016); however, it is unclear how apoptosis of host cells affects IAV production or vRNA replication. We therefore used our live-cell imaging system to examine the apoptotic process following IAV infection. A549(MCP-GFPdN) cells were stained with Hoechst 33342 1 h before IAV infection, and cells were then imaged hourly after infection. We imaged dozens of apoptotic cells. These apoptotic cells initially displayed the typical morphology of epithelial cells anchored to a surface, but then shrank, detached from the plate surface, and formed blebs at Hours 2 and 4 after infection (Figure 3Aa–c); furthermore, nuclei became pyknotic at Hour 2 as nucleus size decreased and chromatin condensed (Figure 3Ad–f). The images of Cell A1 (Figure 3Aa–f) are representative of these findings. In addition, as cells stained with Hoechst dye could be followed by live-cell imaging for only a limited time due to phototoxicity, we proceeded to characterize apoptotic events and PB2-vMSL vRNA punctate signals following IAV infection in the absence of Hoechst dye, and this represents a key advantage afforded by our adapted live-cell imaging system. We found that 74% of cells developed the morphological hallmarks of apoptosis, including cell shrinkage and membrane blebbing (Table 1, Without Treatment group).

**Table 1:** IAV replication in the apoptotic cells.

	Total number of cells	Without replication	With replication	*p* ^a^
**Without treatment**				
Non-apoptotic cells	14 (26%)^b^	14 (26%)	0 (0%)	
Apoptotic cells	39 (74%)	19 (36%)	20 (38%)	<0.001
Total	53			
**Q-VD-OPH treatment** ^c^				
Non-apoptotic cells	51 (65%)	51 (65%)	0 (0%)	
Apoptotic cells	28 (35%)	22 (28%)	6 (8%)	<0.001
Total	79			
**Z-VAD-fmk treatment** ^d^				
Non-apoptotic cells	17 (35%)	17 (35%)	0 (0%)	
Apoptotic cells	31 (65%)	19 (40%)	12 (25%)	<0.001
Total	48			
**Staurosporine** ^e^				
Non-apoptotic cells	6 (16%)	6 (16%)	0 (0%)	
Apoptotic cells	31 (84%)	15 (41%)	16 (43%)	<0.001
Total	37			

a *Value of *p* was calculated by McNemar’s test*.

b *Percentages in the table were calculated by dividing the number with total number of cells, 53, 79, 48, and 37*.

c *A549(MCP-GFPdN) cells were pretreated with 20 μM of Q-VD-OPH for an hour followed by IAV infection and tracked every hour for 24 h under a microscope*.

d *A549(MCP-GFPdN) cells were pretreated with 100 μM of Z-VAD-fmk for an hour followed by IAV infection and tracked every hour for 24 h under a microscope*.

e *A549(MCP-GFPdN) cells were pretreated with 200 nM of staurosporine for an hour followed by IAV infection and tracked every hour for 24 h under a microscope*.

### Characterizing the Fluorescent Signals Observed After IAV Infection

**Figure 4:**
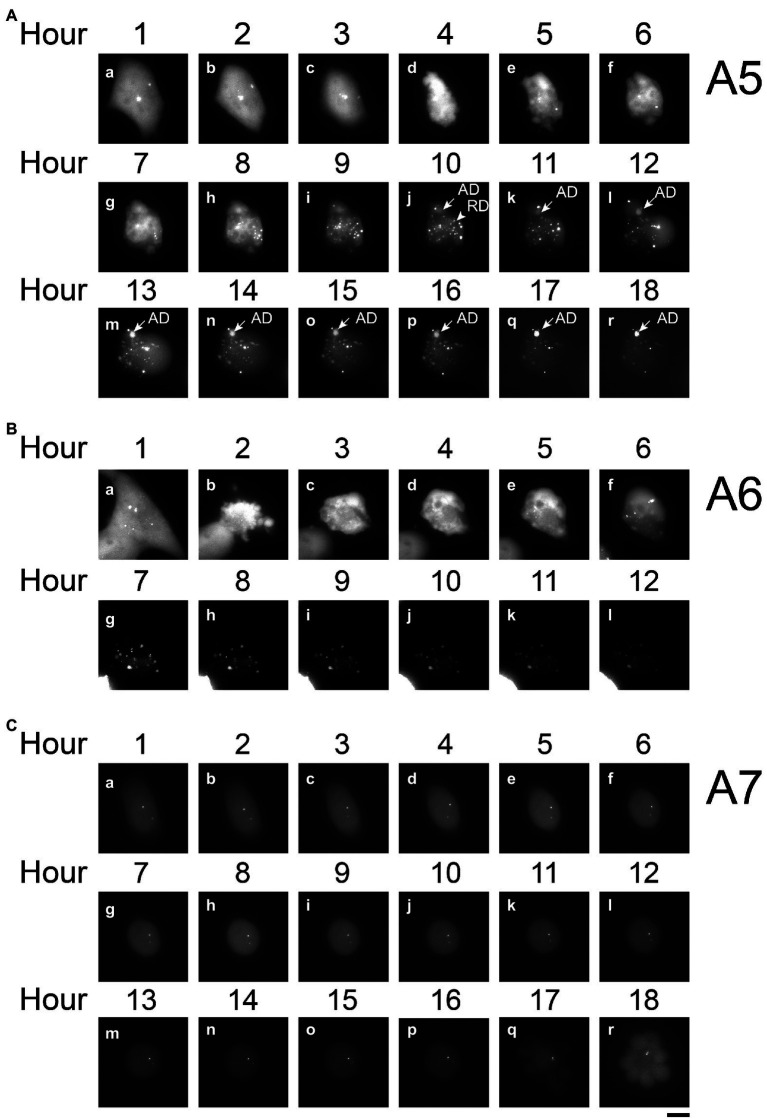
Time-lapse analysis of PB2-vMSL vRNA replication in live cells after IAV infection. A549(MCP-GFPdN) cells were transfected with pHH-PB2-vMSL and infected with two MOI of PR8 IAV at 20 h post-transfection. At 2 h after infection, cells were imaged every hour for 18 h using a Nikon TiE eclipse inverted microscope. RDs (replicated dots) and ADs (apoptosis dots) are indicated by arrows. ADs are primarily static, and are larger in size and have higher signal intensities than RDs. Observations were exemplified by the images captured from representative Cells A5 **(A)**, A6 **(B)**, and A7 **(C)** at the indicated time points, and images are shown as the square of maximum intensity projections across the z-planes in ImageJ. Scale bar: 10 μm.

Since PB2-vMSL vRNA is transcribed from pHH-PB2-vMSL after transfection, it is crucial to distinguish vRNA generated by IAV replication from that transcribed from pHH-PB2-vMSL, so that an accurate assessment of vRNA replication can be made. The signals that arose from PB2-vMSL vRNA transcription were termed transcription dots (TDs), while those that emerged from IAV vRNA replication were termed replicated dots (RDs). We conducted an experiment in which A549(MCP-GFPdN) cells were transfected with pHH-PB2-vMSL. At 16 h post-transfection, cells were imaged every hour for 8 h (Figure 3B). We found that the TD signals were mostly stationary, and generally localized to the same regions over several hours (Figure 3B; representative Cell, A2); moreover, these signals were often composed of multiple PB2-vMSL vRNA speckles, some of which clustered together, while others separated into smaller dots (Figure 3Bb, Hours 1–2) or again clustered together over the next few hours (Figure 3Bb,c, Hours 6–8). These signals were likely present in the nucleolus, as PB2-vMSL vRNA is transcribed from the POL I promoter of pHH-PB2-vMSL, which initiates transcription in the nucleolus (Kong et al., 2011). Furthermore, an earlier study showed that GFP proteins are largely excluded by the nucleolus due to its dense structure (Park et al., 2015). Our imaging results seemed to indicated that the dots were present in the areas where GFP was absent in the nucleus (Supplementary Figure 4), which also implies that TDs are present in the nucleolus. The clustering effect of TDs may be due to lack of viral proteins to enable vRNP nuclear export, and yielded more intense signals as compared to RDs. In addition, TDs mostly disappeared after apoptosis and did not coexist with RDs (Figure 4A, representative Cell, A5, Hours 1–3), and therefore we concluded that TDs are unlikely to interfere with the assessment of RDs derived from vRNA replication.

We further observed a second class of signals that developed during apoptosis following IAV infection, and these were termed apoptotic dots (ADs). To characterize these signals, A549(MCP-GFPdN) cells were infected with IAV at an MOI of 2 and followed hourly for 18 h with live-cell imaging. Images of Cell A3 (Figure 3C) are representative of our findings regarding apoptotic cells, which displayed typical apoptotic changes (Zhang et al., 2018) such as shrinking and blebbing at Hour 7, as well as large high-intensity dots at Hour 10. Over the following hours, the number and intensity of ADs increased, and became prominently visible after Hours 12 and 13 (Figure 3C). ADs are typically stationary, and have substantially higher intensity and larger size than RDs (Figure 3C, Hours 10–18). Treatment of A549(MCP-GFPdN) cells with the apoptosis inducer staurosporine for 10 h revealed that ADs arose as a result of apoptosis, and were independent of IAV infection (Figure 3D). Furthermore, ADs were not stained by 5-ethynyl uridine, a uridine analog that is incorporated into newly synthesized RNA (Supplementary Figure 5), indicating that these dots likely do not contain newly synthesized PB2-vMSL vRNA.

RDs derived from replicated PB2-vMSL vRNA were also examined following IAV infection. A549(MCP-GFPdN) cells were transfected with pHH-PB2-vMSL to express PB2-vMSL vRNA, and then infected with the PR8 strain of IAV at an MOI of 2 at 20 h after transfection. Unlike TDs and ADs, RDs observed after IAV infection displayed rapid and dynamic movement (Figure 3E), and their total number and signal intensity increased exponentially over time. We chose three images from representative Cell A4 (Figure 3E), which had only RDs, to illustrate the dynamics of RDs. The distribution pattern of RDs changed during Hours 3–5; none of the dots were distributed identically in these images (Figure 3E). Moreover, RDs were distributed throughout the cell, as replicated PB2-vMSL vRNAs could be exported to the cytoplasm after IAV infection (Figure 3E). In addition, RDs could be labeled with 5-ethynyl uridine (Supplementary Figure 5), thus verifying the presence of newly synthesized RNA, and enabling us to differentiate between RDs and ADs.

### Apoptosis and vRNA Replication

A549(MCP-GFPdN) cells were transfected with pHH-PB2-vMSL, infected with IAV at an MOI of 2, and tracked by live-cell imaging. Among the 115 cells that were followed, the number of signals and their intensities were measured in 53 of these cells, of which 14 (26%) exhibited neither apoptosis nor PB2-vMSL vRNA replication, 19 (36%) underwent apoptosis but without PB2-vMSL vRNA replication, and 20 (38%) supported both apoptosis and PB2-vMSL vRNA replication. None of the non-apoptotic cells supported detectable IAV replication (Table 1, Without Treatment group).

**Figure 5:**
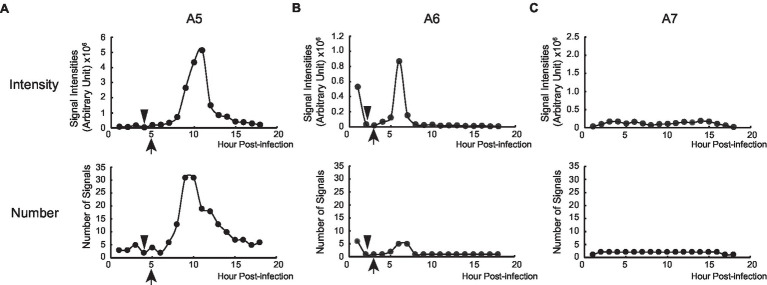
Replication of PB2-vMSL vRNA after IAV infection. The intensity and number of punctate RDs in each z-plane of representative Cells A5 **(A)**, A6 **(B)**, and A7 **(C)** during the entire 18-h imaging period were measured using the Computer-Assisted Plasmid Summation (CAPS) program, and then counted, summed up, and graphed. Triangles indicate when apoptosis was first observed, and arrowheads indicate when vRNA replication was observed to begin.

In cells supporting PB2-vMSL vRNA replication, exemplified by Cell A5 (Figure 4A), several intense signals that remained in the same positions within the first 3 h after infection were noted. These signals resembled those observed in cells that were transfected with pHH-PB2-vMSL but without IAV infection (Figure 3B), and it is likely that these are TDs derived from PB2-vMSL vRNA transcribed from pHH-PB2-vMSL. At Hour 4 (Figure 4Ad), apoptosis became evident, and the simultaneous disappearance of punctate signals was likely due to disassembly of the nucleolus at this apoptotic stage (Soldani et al., 2006). Morphological changes, including size increases of the cell, were observed after Hour 5, when RDs resulting from PB2-vMSL vRNA replication also emerged and became more pronounced after Hour 7 (Figure 4Ae, Hour 5; Supplementary Figure 6a,b). ADs became apparent at Hour 10 (Figure 4Aj), and predominated after Hour 13 (Figure 4Am–r), but the CAPS program (Chiu et al., 2013) was used to exclude these signals from our measurements. Unsurprisingly, some apoptotic cells failed to support vRNA replication (Supplementary Figures 7C, 8C), perhaps because they were infected by defective virus, as most virions in an IAV population are known to be defective (Brooke et al., 2013; Jacobs et al., 2019). It is also possible that signals could not be detected by microscopy due to the absence of pHH-PB2-vMSL. We also identified cells that neither underwent apoptosis nor supported viral genome replication (Figures 4C, 5C).

### Changes of Fluorescent Signal Intensity During Apoptosis

Measuring the intensities of all signals within a representative cell (Cell A5) showed that the sum of signal intensities and number of signals began to increase exponentially starting from Hour 7–11 (Figure 5A; Supplementary Table 2), and subsequently began to decline at Hour 11 post-infection (Figure 5A; Supplementary Table 2). Using the CAPS program to determine Arbitrary Units (AU), we found that in the first 5 h after infection, the total intensity of signals for representative Cell A5 fluctuated between 59,411 and 184,961 AU; however, signal intensity between Hours 6 and 8 increased exponentially, from 221,393 to 353,578 between Hours 6 and 7, and to 744,094 AU between Hours 7 and 8 (Figure 5A; Supplementary Table 2). Between Hours 8 and 9, signal intensity further increased to 2,669,471 AU, and continued to rise until eventually reaching a peak of 5,145,448 AU at Hour 11 (Figure 5A; Supplementary Table 2). As the transcription of PB2-vMSL vRNA from pHH-PB2-vMSL should increase linearly over time, it is likely that the observed exponential rise in signal intensity was driven by vRNA replication; moreover, the rapid decrease in signal intensity seen after Hour 12 (Figure 5A; Supplementary Table 2) suggests that PB2-vMSL vRNA replication only occurred during a short window of about 2–3 h between Hours 8 and 11 post-infection. During this period, RDs were detected in the cytoplasm, as the IAV proteins M1 and NS2 (Lamb and Lai, 1980; Lamb et al., 1981) were present to enable nuclear export of vRNPs; however, given that TDs remained stationary at Hours 1–3 post-infection, but disappeared after apoptosis (Figure 4Aa–c; Supplementary Table 2), it is possible that the IAV proteins enabling vRNP nuclear export were either not expressed prior to apoptosis, or that the enlarged nuclear pores activated by IAV-induced caspase are required to enhance the nuclear export of vRNPs (Muhlbauer et al., 2015).

Replication of PB2-vMSL vRNA in representative Cell A8 followed a similar pattern, except that apoptosis occurred at 6 h post-infection (Supplementary Figure 7A), and RDs became visible between Hours 8 and 9 (Supplementary Figures 6e, 7A), and exponential increases in signal intensity were observed between Hours 8 and 12 (Supplementary Figure 8A). Notably, apoptosis occurred quite early after IAV infection in some cells, and signal intensities in such cells also peaked early; for instance, representative Cell A6 displayed membrane blebbing at Hour 2 after infection (Figure 4B), during which signal intensities declined sharply (Figure 5B), but vRNA replication occurred sooner than in Cells A5 and A8 (Figure 5B; Supplementary Figure 6c,d), with exponential increases observed at Hours 5 and 6 (Figure 5B). A similar pattern was observed for representative Cell A9 (Supplementary Figures 6g,h, 7B, 8B). These findings show that apoptosis can occur at an earlier (Hours 3–4) or later (Hours 5–7) time post-infection, but RDs, indicative of vRNA replication, consistently appear about 2 h after apoptosis initiation, following which exponential growth in signal intensity or number is observed (Figure 5; Supplementary Figure 8). This suggests that IAV vRNA replication does not randomly proceed in infected cells, but may be programmed to take advantage of host cell apoptotic processes to achieve exponential replication. Importantly, these findings indicate that vRNA replication is initiated asynchronously in an infected cell population, and that replication consistently begins in the early stages of apoptosis; moreover, vRNA replication was observed to occur only in apoptotic cells.

### Effect of Apoptosis Inhibitors on vRNA Replication

**Figure 6:**
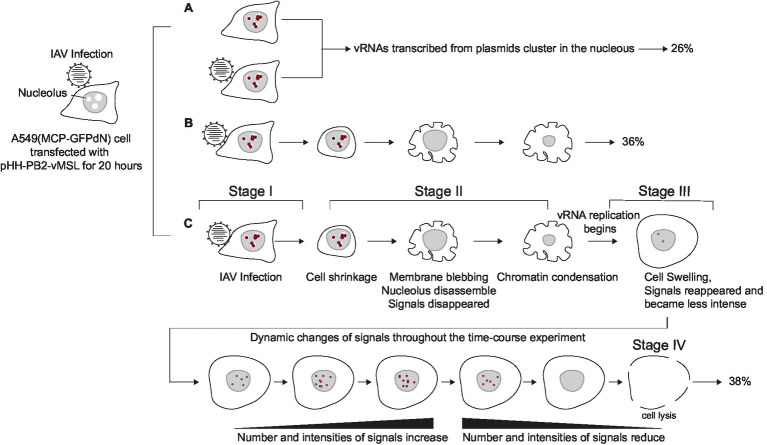
Timing of IAV-induced apoptosis and PB2-vMSL vRNA replication. A549(MCP-GFPdN) cells transfected with pHH-PB2-vMSL were infected by IAV and then imaged for 18 h. The images revealed three types of cells with punctate signals: **(A)** non-apoptotic cells; **(B)** cells that underwent apoptosis but failed to support PB2-vMSL vRNA replication; and **(C)** cells that initiated apoptosis and also supported PB2-vMSL vRNA replication. The results also showed that PB2-vMSL vRNA replication occurred soon after membrane blebbing and chromatin condensation.

Notably, increased vRNA signal intensities were only observed in cells that underwent apoptosis (Figures 4–6; Supplementary Figures 7, 8; Table 1), and we proceeded to ascertain whether apoptosis was key to vRNA replication by treating cells with two pan-caspase inhibitors, Q-VD-OPH and Z-VAD-fmk. Z-VAD-fmk increased the percentage of non-apoptotic cells from 26% to 35%, while Q-VD-OPH, a stronger apoptosis inhibitor than Z-VAD-fmk (Caserta et al., 2003; Keoni and Brown, 2015), increased the percentage of non-apoptotic cells to 65% (Table 1). Subsequently, the percentage of cells with vRNA replication decreased from 38% to 25% in cells treated with Z-VAD-fmk, and declined further to 8% in Q-VD-OPH-treated cells (Table 1). We proceeded to conduct a randomized assessment of the number and intensity of PB2-vMSL vRNA signals in A549(MCP-GFPdN) cells infected by PR8 IAV, and live-cell imaging results showed that Q-VD-OPH reduced both the total number and intensity of RDs (Table 2), indicating that Q-VD-OPH inhibits vRNA replication. Conversely, treatment with the apoptosis inducer staurosporine increased the percentage of apoptotic cells from 74% to 84%, while moderately elevating the percentage of cells with RDs from 38% to 43% (Table 1). The results suggest that IAV-induced apoptosis may be required or is already sufficient to support IAV vRNA replication, and the addition of other apoptosis inducers is therefore unable to further enhance the process (Laghlali et al., 2020). It is also possible that other factors such as the availability of cellular resources or the limits of cellular machinery may place a cap on viral propagation. Importantly, these findings indicate that vRNA replication is initiated asynchronously in an infected cell population, and that replication consistently begins in the early stages of apoptosis; moreover, vRNA replication was observed to occur only in apoptotic cells.

**Table 2:** Inhibition of vRNA replication by an apoptosis inhibitor.^a^^,^^d^

	# of RDs	Intensities of RDs (A.U.)^b^
**Without treatment**		
	10	481,596
5	315,734
16	2,081,425
22	980,049
25	1,862,408
17	1,104,384
Sum	95	6,825,596
**Q-VD-OPH treatment** ^c^		
	6	199,249
5	154,952
14	356,015
5	191,817
6	232,880
10	365,936
Sum	46	1,500,849

a *Total number and intensity of signals containing replicated dots (RDs) in cells were determined as their replication reached the peak*.

b *Arbitrary Unit (A.U.) of signals was determined by the Computer-Assisted Plasmid Summation (CAPS) program*.

c *A549(MCP-GFPdN) cells were pretreated with 20 μM of Q-VD-OPH for an hour followed by IAV infection, and then tracked every hour for 24 h under a microscope*.

d *Value of *p* between the sum of without treatment and Q-VD-OPH treatment was less than 0.05 as determined by Student’s *t*-test*.

### Generating IAV Virions Carrying PB2-vMSL vRNA

To ascertain whether PB2-vMSL vRNA is encapsulated into viral particles, we cotransfected HEK293T cells with pQCXIH-PB2 (encoding PB2) and all the pDZ reverse-genetics plasmids, except for pDZ-PB2, which was replaced with pHH21 (an empty vector), pHH-PB2, or pHH-PB2-vMSL, to, respectively, produce the IAV derivatives, IAV(null), IAV(PB2), or IAV(PB2-vMSL). Considering that ectopically expressed PB2 in MDCK cells can support IAV replication, we theorized that IAV(PB2) virions would also contain PB2 vRNA. It is well known that incomplete sets of vRNA can be packaged into virions (Brooke et al., 2013; Jacobs et al., 2019) and thus IAV(null) virions should contain IAV vRNA but without PB2 vRNA; however, it is not known whether PB2-vMSL vRNA was packaged in the IAV(PB2-vMSL) virions. We proceeded to harvest virus from the culture medium (P0) of transfected HEK293T cells at 3 days post-transfection, and used it to infect MDCK(PB2) cells, an MDCK(MCP-GFPdN) cell line transduced to express PB2 in order to amplify the virus (P1). At 3 days after infection, 10% of P1 virions were then used to infect MDCK(PB2) cells to generate P2 virions. These were then harvested, and vRNA was extracted and assessed for PB2 vRNA using RT-qPCR. Compared to cells infected by IAV(null) viruses, in cells infected by IAV(PB2) viruses, copies of PB2 vRNA in P1 virions released into the culture medium were 28-fold higher, and were 205-fold higher in P2 virions (Supplementary Table 3). In cells infected by IAV(PB2-vMSL) P0 virions, a 66-fold increase of PB2 vRNA was observed for P1 virions, and a 246-fold increase was observed for P2 virions (Supplementary Table 3). The results confirm that PB2-vMSL vRNA is indeed packaged into IAV virions after infection.

### Imaging vRNA in MDCK Cells After Infection

**Figure 7:**
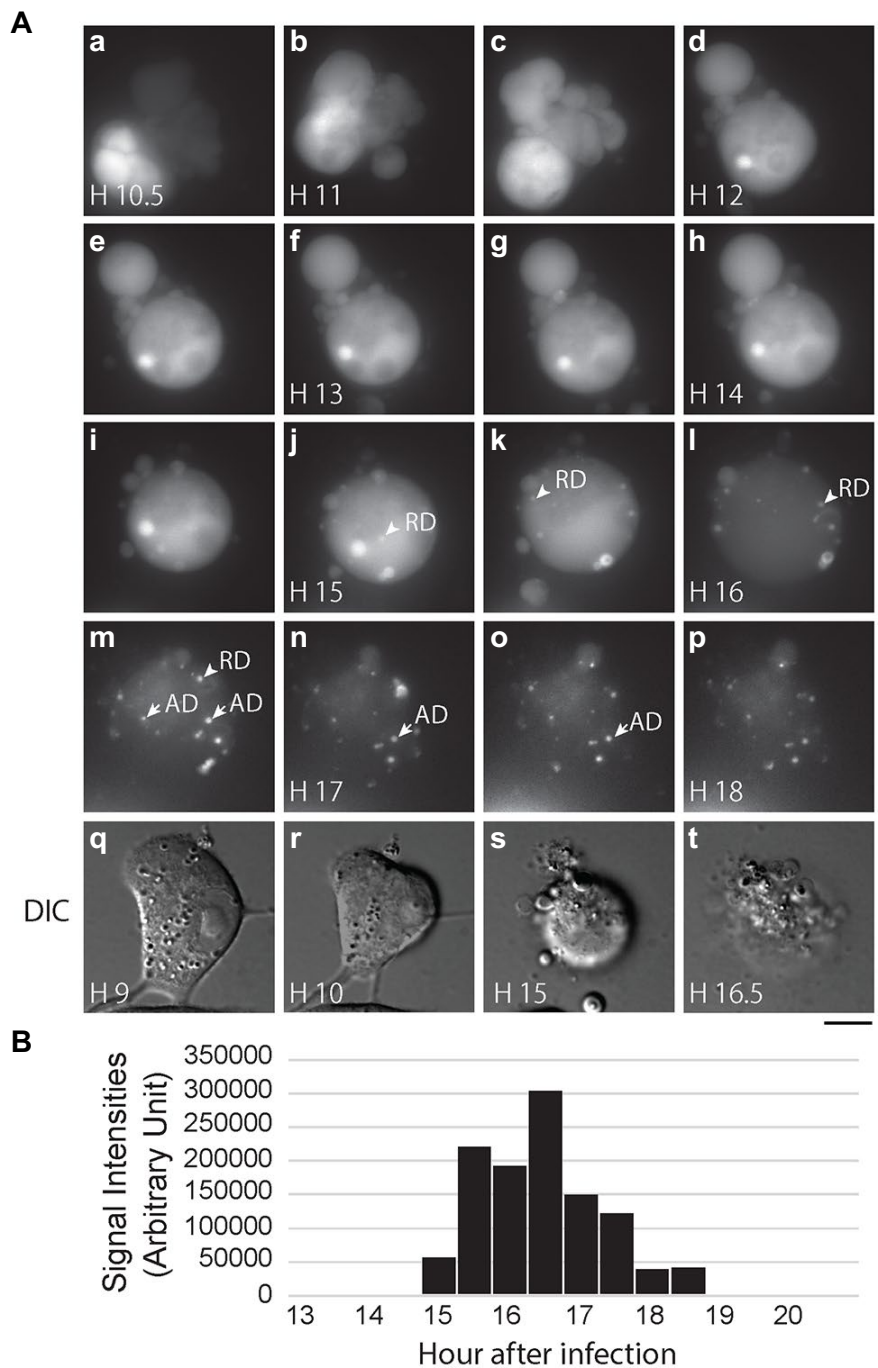
IAV vRNA replication in MDCK cells after infection. **(A)** MDCK(PB2) cells were infected with IAV(PB2-vMSL) virus at an MOI of 10. After infection, cells were imaged every 30 min for 18 h under a Nikon TiE eclipse inverted microscope. RDs (replicated dots) and ADs (apoptosis dots) are indicated by arrows. ADs are primarily static, and are larger in size and have higher signal intensities than RDs. The images derived from representative cells at the indicated time points are shown as the square of maximum intensity projections across the z-planes in ImageJ. Scale bar: 10 μm. **(B)** Intensity of punctate signals in each z-plane during the entire 18-h imaging period was measured using the CAPS program, and then counted, summed up, and graphed.

After confirming that PB2-vMSL vRNA is packaged into IAV virions, we infected MDCK(PB2) cells with IAV(PB2-vMSL) at an MOI of 10; at this MOI, almost all infected cells become apoptotic. We randomly selected 12 cells to track with live-cell imaging, of which nine eventually exhibited RDs. Notably, no RD signals were observed in these cells prior to the onset of apoptosis. Representative images of our findings were captured using Cell M6A: at Hour 10.5 after infection, apoptosis was evident, as indicated by chromatin condensation (Figure 7Aa,q–r), and between Hours 15 and 16, RD signals appeared (Figure 7Aj–l). Unlike the ADs that emerged between Hours 16.5 and 18 (Figure 7Am–p), RDs did not remain stationary. The total intensity of RD signals increased from Hour 15, reached a peak at Hour 16.5, and gradually decreased thereafter (Figure 7B), exhibiting a similar pattern as that observed in experiments with transfection systems (Figure 5; Supplementary Figure 8). Additionally, during our CAPS measurement of nine cells out of 12 cells with vRNA replication in MDCK(PB2) cells, we found that RDs appeared about 4–5 h after apoptosis started, which is longer than the 1–2 h observed in A549 cells after transfection and infection.

### Replication of vRNA in Apoptotic Cells

**Figure 8:**
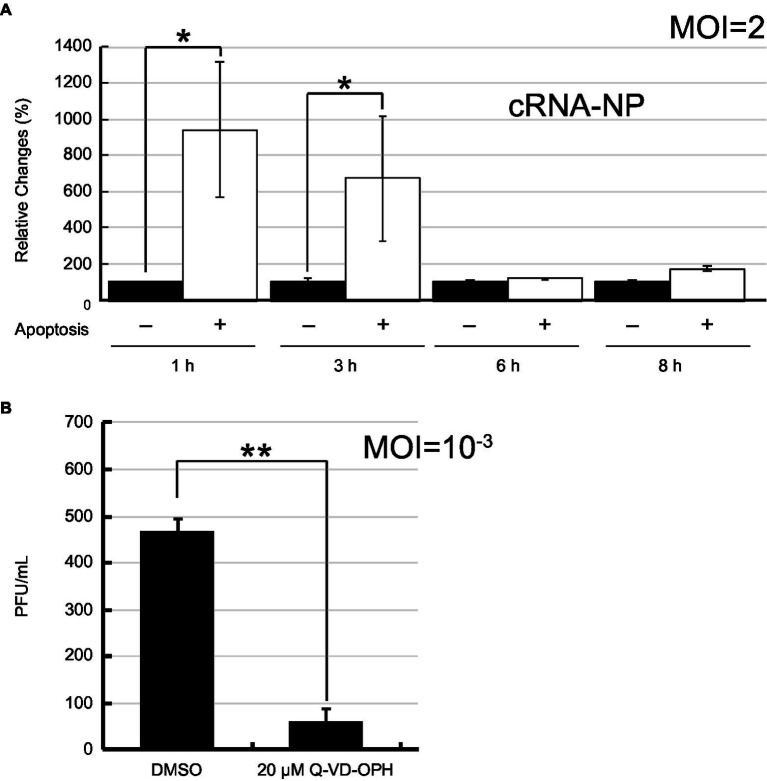
IAV replication in apoptotic cells after infection. **(A)** A549 cells were infected with PR8 at an MOI of 2. Apoptotic (+) and non-apoptotic (−) cells were sorted by flow cytometry after Annexin V staining at the indicated time points post-infection. After isolation of total RNA from the cells, 18S rRNA and NP cRNA (cNP) were quantified by RT-qPCR, after which cRNA levels were normalized to levels of 18S rRNA. The amount of cRNA in non-apoptotic cells was set to 100%. **(B)** A549 cells were infected with IAV PR8 virus at an MOI of 0.001 and treated with Q-VD-OPH for 24 h. IAV released from cells was collected and quantified by plaque assay. The SD was calculated from two independent sets of experiments. The results were analyzed statistically with Student’s *t*-test. ** and * represent statistical significance of <0.01 and <0.05, respectively.

To demonstrate that IAV vRNA preferentially replicated in apoptotic cells as PB2-vMSL vRNA, we infected A549 cells with PR8 strain of IAV and stained cells with annexin V (AV) after infection. After sorting apoptotic cells from the population, we determined the amounts of vRNA in apoptotic cells by RT-qPCR. As IAV virions usually do not contain a complete set of vRNA and may not replicate (Brooke et al., 2013; Jacobs et al., 2019), the presence of the vRNA from the defective viruses after infection can increase the background to our assessment. To circumvent the problem, the amounts of NP cRNA (cNP), which are derived only from cells with vRNA replication, was determined by RT-qPCR, and normalized them to the amount of 18S rRNA. Accordingly, we found that at Hour 1 after infection, the amount of cNP in apoptotic cells was 9.4-fold higher than that in non-apoptotic cells on a per cell basis (Figure 8A). The amount of cNP decreased as apoptosis progressed; a 6.7-fold increase was observed at Hour 3. However, the difference was no longer observed after 6 h post-infection (Figure 8A). According to our study, we found that PB2-vMSL vRNA replicates within a short period of 2–3 h; after this period, the intensity of PB2-vMSL vRNA signal decreases rapidly to a background level (Figure 5; Supplementary Figure 8). If replication of vRNA from PR8 follows the same dynamic pattern, the cells may not degrade its vRNA during the first 3 h of infection, explaining why accumulation of vRNA on a per cell basis was observed (Figure 8A). After 3 h of infection, it is likely that vRNA that replicated starting at Hour 1 may cease to replicate and decrease to a low level, resulting in an increase of the numbers of apoptotic cells that lost their vRNA. As replication of vRNA is asynchronous, although some cells may continue to initiate vRNA replication at a given time, vRNA continued to lose from the cells that had vRNA replication, resulting in an increase of a population of apoptotic cells without vRNA, which may explain why the ratio between vRNA and 18S rRNA decreased after infection after Hour 6 (Figure 8A).

Finally, we infected A549 cells with PR8 virus at an MOI of 10^−3^ and treated the cells with a pan-caspase inhibitor, Q-VD-OPH. The progeny virus produced from cells was enumerated by plaque assays at 24 h post-infection. We found that the viral production reduced by 7.8-fold in the presence of apoptosis inhibitor (Figure 8B), illustrating the contribution made by apoptosis to IAV replication.

## Discussion

In this study, we describe the establishment of a robust live-cell imaging approach to monitor IAV vRNA replication. First, we constructed a pHH-PB2-vMSL plasmid, which allows transcription of PB2-vMSL vRNA from a POL I promoter. The binding of MCP-GFPdN to the MSL structure in the PB2-vMSL vRNA enabled the microscopic visualization of fluorescent punctate signals (Figures 1, 2). After the cells are transfected with all pDZ plasmids supporting IAV reverse genetics or after infection, further allowed us to observe the replication of PB2-vMSL vRNA (Figure 4; Supplementary Figure 7). We also found that PB2-vMSL vRNA is packaged into IAV virions (Supplementary Table 3), showing that the expression of PB2-vMSL vRNA has little effect on IAV replication; the dynamic movement of the signals reflects truly the behavior of IAV vRNA. This live-cell imaging approach can be used to track IAV vRNA replication in single cells, without the disadvantages of short fluorophore half-life or interference with viral proteins, which have limited the utility of previous live-cell imaging systems (Lakadamyali et al., 2003; Chen and Zhuang, 2008; Li et al., 2010).

During the live-cell imaging process, we identified three types of punctate signals in the cells: TDs, RDs, and ADs. Unlike TDs and ADs, RDs are highly dynamic, they are present at one time point and then replaced by a new set of dots at the next hours (Figure 3E). Additionally, the intensity of RDs increases exponentially after apoptosis (Figure 5; Supplementary Figure 8) and the dots contains newly synthesized RNA (Supplementary Figure 5); only RNA replication can result in such an increase and synthesis. Although TDs and ADs have intensity higher than RDs, neither the intensity nor the size of the dots was used as a criterion for distinguishing RDs from TDs and ADs. As TDs do not coexist with RDs and disappear following the advent of apoptosis and prior to the appearance of RDs (Figure 4A, Hour 4), they do not pose any problems for the measurement of RDs. However, ADs appear and coexist with RDs during the latter stages of vRNA replication (Figures 3, 4, Hours 10–12; Supplementary Figure 7), and may affect the accurate assessment of vRNA replication if not adequately differentiated. As ADs dots are stationary and remain in a cell even after vRNA replication ends, the distribution pattern of these dots after PB2-vMSL vRNA replication could be used as a reference (Figure 4A, Hours 13–18), allowing us to identify the ADs in a cell when ADs and RDs coexist (Figure 4A, Hours 10–12). To expedite and accurately measure the intensity of RDs, we first scanned all the z-planes of a cell with the CAPS program prior to the measurement of signal intensity. Images with signals were collected and compared with those in the same z-planes over the next hours, and were eventually compiled together to derive a time-based record of signal tracking. After RDs were identified, the intensity of RDs in different z-planes was measured manually using CAPS.

The reporter PB2-vMSL vRNA used in this system not only replicates in A549 cells in a transfection-infection system (Figures 4, 5; Supplementary Figures 7, 8), but also replicates in MDCK(PB2) cells after infection of IAV(PB2-vMSL) virus (Figure 7). In the transfection-infection system, RD signals emerged 1–2 h after the disappearance of apoptotic blebs (Figure 4A, Hours 4 and 5), and in an MDCK infection system, the total intensity of RDs started about 4 h after emergence and peaked at about 1.5 h after emergence (Figure 7). We subsequently observed an abrupt decrease of RD signals in both systems after peak intensity was reached (Figures 4, 5, 7; Supplementary Figures 7, 8). The similar patterns of RD signal changes observed in both the transfection-infection and infection systems indicate that these were unlikely to be caused by artifacts in either system, and demonstrate that the observed apoptosis in host cells was likely driven by IAV infection and not transfection or other experimental processes. Furthermore, we observed that the percentage of apoptotic cells in mock infected controls at 8 h after infection was about 5%, and this is comparable to levels described for A549 cells in the literature (Ying et al., 2015). In addition, the microscope used in our live-cell imaging method employs a light-emitting diode (LED) light source, which is considered to be less harsh on cells than ultraviolet or laser light sources (Tosheva et al., 2020). From imaging data of cell cultures, we observed that at 2 h after infection, apoptosis rates in mock infected A549 cells were about 1.28%, compared to 9.38% for IAV-infected cells; and at 4 h after infection, mock infected cells had apoptosis rates of 1.71%, compared to rates of 21.95% in IAV-infected cells. This demonstrates that apoptosis was likely not caused by handling and imaging methods. We also showed that an apoptotic inducer did not increase vRNA replication significantly (Table 1), and this suggests that imaging-induced apoptosis may not be an important influencing factor on vRNA replication observed in this study.

IAV infection is known to induce apoptosis in host cells (Schultz-Cherry and Hinshaw, 1996; Morris et al., 1999; Schultz-Cherry et al., 2001; Zamarin et al., 2005; Halder et al., 2011; Muramoto et al., 2013; Gao et al., 2015; Nailwal et al., 2015; Nacken et al., 2017; Pasricha et al., 2018), but the role of apoptosis in the IAV infection cycle is not fully understood as yet. Previous studies have shown that apoptosis can serve as a host defense mechanism against viral infection (Herold et al., 2012; Atkin-Smith et al., 2018), but other studies indicate that apoptosis can enhance viral replication and dissemination while also limiting the pro-inflammatory immune response to infection (Wurzer et al., 2003, 2004). However, a view is emerging that IAV-induced apoptosis, as opposed to that initiated by cellular or host immune processes, may act to facilitate viral replication and propagation (Atkin-Smith et al., 2018; Ning et al., 2019); for instance, it is known that ectopic overexpression of Bcl-2 restricts IAV-induced apoptosis and IAV replication in MDCK cells, but knockdown of Bak, a downstream member of the Bcl-2 signaling pathway, subsequently led to significant increases in IAV-induced cell death and viral replication in mouse embryonic fibroblasts (McLean et al., 2009). Conversely, knockdown of the apoptotic inducer Bax blocked apoptosis induction and led to retention of IAV NP in cell nuclei, while host cells were driven to an alternative autophagy pathway of cell death (McLean et al., 2009). A recent study has also shown that the apoptosis inducer and anti-inflammatory regulator Annexin-A1 can enhance IAV infectivity, IAV-mediated apoptosis, and viral replication, while ANXA1-deficient mice exhibit a survival advantage following IAV infection (Arora et al., 2016). In this study, our live-cell imaging system revealed that of 53 cells examined after IAV infection, 74% initiated apoptosis, with 38% of all cells undergoing apoptosis while also supporting IAV replication (Table 1). Of the 36% of cells that initiated apoptosis but failed to support IAV replication (Table 1), it is likely that these were infected by defective viral particles (Brooke et al., 2013; Jacobs et al., 2019). We also found similar phenomenon in apoptotic and non-apoptotic cell populations isolated by cell sorter (Figure 8A), showing that vRNA replication occurs preferentially in apoptotic cells. Moreover, treatment with a pan-caspase inhibitor, Q-VD-OPH, subsequently reduced the number of apoptotic cells (Figure 8B). These findings are consistent with previous observations that caspase 3 activity is essential for efficient influenza virus propagation (Wurzer et al., 2003, 2004; Ning et al., 2019). Our results support the view that IAV-induced apoptosis in host cells plays a crucial role in viral replication and propagation. To address the possibility that non-apoptotic cells were all uninfected by PR8 IAV, and therefore vRNA replication would not be observed, we separated non-apoptotic and apoptotic cells by cell sorting, and RNAseq data showed that IAV mRNA is detected in both cell populations (Supplementary Figure 9), demonstrating that the lack of vRNA replication in non-apoptotic cells cannot be attributed to the absence of IAV infection.

Since our live-cell imaging system allows for near real-time monitoring of time-sensitive processes, an advantage over previous biochemical or immunological methods, we further investigated the timing of IAV-induced apoptosis and vRNA replication, and noted that the initiation and progression of these processes varies among individual infected cells. For some cells, apoptosis induction was observed as early as 2–4 h after IAV infection (Figure 4). In previous IAV studies, apoptosis induction was detected as early as 4 h post-infection in IAV-infected A549 cells, and increased until 24 h post-infection (Tripathi et al., 2013); while another study showed that at about 2 h post-infection with IAV, death receptor pathway genes were found to be significantly upregulated in human bronchial epithelial cells (Othumpangat et al., 2018). These studies suggest that apoptosis induction can indeed occur quite early after IAV infection, but this is admittedly difficult to observe using biochemical or immunological techniques. Further studies to ascertain this using real-time or single-cell laboratory methods are warranted. The live-cell imaging method used in this study also allows for much more sensitive detection of apoptosis, since observation at the cellular level is possible, and this demonstrates a key advantage of this system. Importantly, we observed that vRNA replication consistently occurs about 2 h following apoptosis induction in transfected or infected A549 cells (Figure 5; Supplementary Figure 8), and 4–5 h after apoptosis occurs in MDCK(PB2) cells infected with IAV(PB2-vMSL) (Figure 7A), suggesting that vRNA replication is programmed to take advantage of host cell apoptotic processes. For example, in the representative Cells A6 and A9, replication occurred early and reached a peak at Hours 6 and 7, respectively (Figures 4B, 5B; Supplementary Figures 7B, 8B). However, replication and peak signal intensity occurred at later time points after infection in representative Cells A5 and A8 (Figures 4A, 5A; Supplementary Figures 7A, 8A). Our studies clearly show that a dynamic process marked by a series of morphological changes occurs between IAV infection and vRNA replication in IAV-infected cells (Figure 6). At Stage I after IAV infection, cells initially have a morphology typical of epithelial cells (Figure 4A, Hour 1; Figure 4B, Hour 1; Figure 6). In Stage II, apoptosis becomes evident with cell shrinking (Figure 4A, Hour 4) or blebbing (Figure 4B, Hour 2; Figure 6). Subsequently in Stage III, cells swell (Figure 4A, Hour 6; Figure 4B, Hour 3; Figure 6), and most importantly, vRNA replication is observed at this swelling stage. This dynamic process was observed in all tracked cells with vRNA replication without exception, indicating that vRNA replication occurs after apoptosis. This conforms with the results of previous studies (Tran et al., 2013; Yeganeh et al., 2018) that have demonstrated the key importance of host cell apoptosis to IAV replication. Notably, several recent imaging studies of vRNA replication in epithelial cell cultures (Lakdawala et al., 2014; Chou and Lionnet, 2018; Haralampiev et al., 2020; Shah et al., 2020) did not use apoptosis markers to assess cells for apoptotic status, but their imaged cells lacked epithelial morphology and resembled Stage III morphology of IAV-infected cells (Figure 6) in our study. It is possible that the cells in these recent imaging studies had already passed the cell shrinkage and blebbing stages to enter the stage of heightened vRNA replication, but without apoptosis markers and time-tracking of cell morphology changes, this cannot be ascertained, and further studies are warranted.

**Figure 9:**
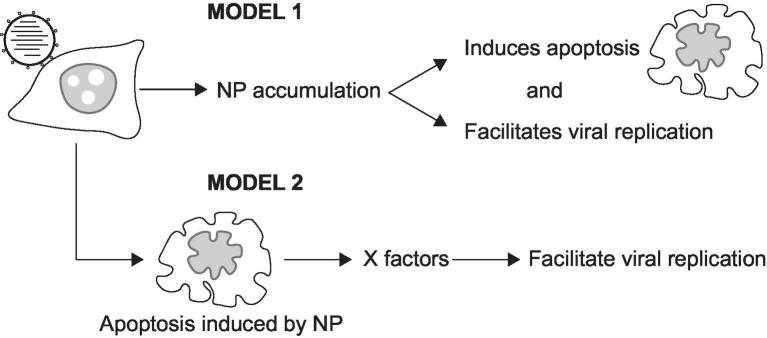
Models illustrating the involvement of NP in apoptosis and vRNA replication. NP is known to induce both apoptosis and vRNA replication. Model 1 shows that the accumulation of NP may act to induce vRNA replication at a specific stage of apoptosis. Model 2 shows that cellular factors (X factors) expressed during apoptosis may act to facilitate vRNA replication.

Our temporal measurements demonstrate that IAV vRNA replication is asynchronous in infected cells (Figures 4, 5; Supplementary Figures 7, 8). As IAV infection is known to affect cell cycle progression (He et al., 2010), the possibility that the asynchronicity of replication initiation and peaking of replication signals may reflect IAV infection of cells at different stages of the cell cycle is worthy of further investigation. Another possibility lies in the observation that PB2-vMSL vRNA replication occurs only after membrane blebbing and chromatin condensation (Figures 4, 6; Supplementary Figure 7); as it is known that several IAV proteins can induce apoptosis during infection (Schultz-Cherry and Hinshaw, 1996; Morris et al., 1999; Schultz-Cherry et al., 2001; Zamarin et al., 2005; Halder et al., 2011; Muramoto et al., 2013; Gao et al., 2015; Nailwal et al., 2015; Nacken et al., 2017; Pasricha et al., 2018; Yeganeh et al., 2018), among which NP has been implicated in both apoptosis induction and vRNA replication (Nailwal et al., 2015), it is possible that NP or other viral proteins may need to accumulate to a certain threshold after infection in order to induce apoptosis and vRNA replication, which may occur simultaneously (Nailwal et al., 2015). There may also be other host factors induced during apoptosis that contribute to the enhancement of vRNA replication (Figure 9). In addition, our observation of replication signal intensity peaking conforms with previous studies that show maximal levels of vRNA replication at 10–12 h post-infection (Robb et al., 2009; Phan et al., 2021), and we further found that the intensities of these signals declined soon after peaking (Figure 5; Supplementary Figure 8; Supplementary Table 2). Interestingly, a recent study of another RNA virus, human coronavirus OC43, also found that coronavirus-induced apoptosis in Vero cells and MRC-5 cells was important for viral replication. Coronavirus-induced apoptosis was similarly dependent on caspase 3, and could induce cell cycle arrest at the S and G2/M phases; moreover, treatment with the pan-caspase inhibitor Z-VAD-fmk also reduced host cell apoptosis and coronavirus replication (Maharjan et al., 2021). It is known that replication in apoptotic cells can help viruses to avoid the scrutiny of the immune system, and can also facilitate the release of viral particles (Maharjan et al., 2021), so it is no surprise that viruses may act to hijack host cell apoptotic processes. These findings also suggest that caution may be needed with proposed antiviral approaches that seek to promote apoptosis in host cells to counter viral propagation (Atkin-Smith et al., 2018; Downey et al., 2018).

Although IAV vRNA replication has been studied extensively in the past, this study offers a novel near real-time perspective of how IAV replication occurs in live cells, and provides important insight into IAV physiological processes, which cannot be obtained from studying a population of IAV-infected cells using biochemical or immunological methods. Our approach offers a way to visualize the kinetics of IAV replication after viral infection, and this live-cell imaging system of IAV infection can differentiate between the signals caused by transcription, vRNA replication, and apoptosis. Spatial and temporal measurements enabled by this system demonstrate that replication occurs only in apoptotic cells at a stage after membrane blebbing and chromatin condensation. Live-cell imaging also revealed the dynamic movement of replication products in infected cells. Together, these results illustrate the utility and potential of this approach in exploring viral processes and viral-host interactions in individual cells infected with IAV. Further research employing this system to assess the interaction between IAV infection, host cell apoptosis, and the effect of antiviral nucleoside analogue drugs, such as fabiravir or ribavirin, is being planned.

## Conclusion

In this study, we established an adapted live-cell imaging system to facilitate the observation of time-sensitive processes pertaining to IAV replication and apoptosis in infected host cells. We found that IAV replicates asynchronously after infection in apoptotic cells, but not in non-apoptotic cells, and both transfection and infection systems demonstrated similar vRNA replication patterns that consistently occurred within a specific time range after apoptosis induction. Moreover, treatment with apoptosis inhibitors reduced IAV vRNA replication and viral production. These time-sensitive results could only have been observed using a live-cell imaging system, and suggest that IAV can exploit host cell apoptosis to facilitate vRNA replication and viral propagation.

## Data Availability Statement

The datasets presented in this study can be found in online repositories. The names of the repository/repositories and accession number(s) can be found at: https://www.ncbi.nlm.nih.gov/sra/PRJNA786297.

## Author Contributions

Y-FC: conceptualization, software, data curation, writing—original draft preparation, supervision, and project administration. Y-FC, Y-WH, C-YC, and Y-CC: methodology and investigation. Y-FC, Y-WH, and Y-CC: validation and visualization. Y-FC, Y-NG, and Y-WH: formal analysis. Y-FC, R-LK, and S-RS: resources. Y-FC and R-LK: writing—review and editing. Y-FC, C-GH, and S-RS: funding acquisition. All authors contributed to the article and approved the submitted version.

## Funding

This work was financially supported by the Ministry of Science and Technology, Taiwan (MOST-105-2320-B-182-033, MOST106-2320-B-182-036-MY3, and MOST 109-2320-B-182-028-MY3); the National Health Research Institute (NHRI-EX108-10623BI); the Chang Gung Medical Research Program (CMRPD1G0511, CMRPD1G0512, CMRPD1K0321, and CMRPD1K0322); Chang Gung Memorial Hospital, Linkou (BMRPF14); and the Research Center for Emerging Viral Infections from The Featured Areas Research Center Program within the framework of the Higher Education Sprout Project by the Ministry of Education (MOE) and MOST in Taiwan (MOST 110-2634-F-182-001 and MOST 109-2327-B-182-002).

## Conflict of Interest

The authors declare that the research was conducted in the absence of any commercial or financial relationships that could be construed as a potential conflict of interest.

The reviewer H-CY declared a shared affiliation with one of the authors, Y-CC, to the handling editor at the time of review.

## Publisher’s Note

All claims expressed in this article are solely those of the authors and do not necessarily represent those of their affiliated organizations, or those of the publisher, the editors and the reviewers. Any product that may be evaluated in this article, or claim that may be made by its manufacturer, is not guaranteed or endorsed by the publisher.

## Footnotes

^1^ https://www.ncbi.nlm.nih.gov/sra/PRJNA786297
