# Supplementary material for: Visualizing Influenza A Virus vRNA Replication
Source: Front Microbiol. 2022 Jun 6;13:812711. doi: 10.3389/fmicb.2022.812711 (PMC9207383; doi:10.3389/fmicb.2022.812711)
Supplement: Supplementary file 1 [file Data_Sheet_1.pdf]

Supplementary Figures and Tables

Supplementary Figure 1. Replication efficiency of PB2-vMSL vRNA in HEK293T cells. HEK393T cells were cotransfected with pHH-PB2 (A) or pHH-PB2-vMSL (B) with (filled column) or without (empty column) plasmids encoding RdRp and NP. Total RNA was isolated with TRIzol reagent at 24 h post-transfection and reverse-transcribed to cDNA with specific PB2 primer targeting PB2 vRNA or Oligo dT for GAPDH. The cDNA of PB2 vRNA and PB2-vMSL was then quantified by qPCR and normalized with the amount of GAPDH mRNA. The relative amount of cDNA from cells without being cotransfected with RdRp and NP plasmids was set as 1 to determine the fold changes. The experiment was repeated three times and data were analyzed by Student's t test.

Supplementary Figure 2. Control experiment showing the lack of punctate signals after transfecting individual plasmids. HEK293T cells were cotransfected with pMCP-GFPdN (A) or pMCP-GFP (B); individual plasmid encoding IAV proteins PB1 (a), PB2 (b), PA (b), NP (d), M proteins (e), NA (f), HA (g), NS proteins (h); empty vector (m and n), pHH-PB2 (i), pHH-PB2-vMSL (k), pHH-PB2-mMSL (o); (j, l, p) pHH-PB2 (j), pHH-PB2-vMSL (l), pHH-PB2-mMSL (p), and plasmids encoding RdRP and NP. Cells were fixed at 16 h after transfection. The images of representative cells were acquired under a Nikon TiE eclipse inverted microscope and are shown as the square of maximum intensity projections across the z-planes in ImageJ. Scale bar: 10 μm.

Supplementary Figure 3. Expression of NP in A549(MCP-GFPdN) cells after transfection with pHH-PB2-vMSL vRNA and infection with PR8 IAV. A549(MCP-GFPdN) cells were transfected with pHH-PB2-vMSL and infected with 2 MOI of PR8 IAV at 20 h post-transfection. Cells were fixed at 6 h post-infection with paraformaldehyde, and immunostained with rabbit anti-NP antibody and goat anti-rabbit IgG conjugated with Alexa Fluor 594. The images of representative cells were acquired using a Nikon TiE eclipse inverted microscope and are shown as the square of maximum intensity projections across the z-planes in ImageJ. Scale bar: 10 μm.

Supplementary Figure 4. Distributions of PB2-vMSL vRNA transcribed from the POL I promoter of pHH-PB2-vMSL. A549(MCP-GFPdN) cells were transfected with pHH-PB2-vMSL, and at 16 h after transfection, cells were imaged every hour for 8 h. The PB2-vMSL vRNA signals (left) and DIC images (right) at hour 5 post-infection in the representative Cell A2 (Figure 3B) were shown. The nucleus and nucleolus were annulated with dashed lines.The arrowheads indicate the signals derived from PB2-vMSL vRNA. Images were acquired using a Nikon TiE eclipse inverted microscope and are shown as the square of maximum intensity projections across the z-planes in ImageJ. Scale bar: 10 μm.

Supplementary Figure 5. **Labeling newly synthesized vRNA in A549(MCP-GFPdN) cells.** A549(MCP-GFPdN) cells were transfected with pHH-PB2-vMSL and infected with 2 MOI of PR8 IAV at 20 h post-transfection. Infected cells were (a-l) treated for 8 h with 1 mM 5-ethynyl uridine (EU). Cells were fixed with paraformaldehyde, and newly synthesized RNA in EU-labeled cells were then detected using Click-iT chemistry. Cells with (a-d) RDs and ADs; (e-h) ADs but without RDs; or (i-l) neither ADs nor RDs were imaged. An AD in c is circled to show its size. The images of representative cells were acquired using a Nikon TiE eclipse inverted microscope and are shown as the square of maximum intensity projections across the z-planes in ImageJ. Scale bar: 10 µm.

Supplementary Figure 6. Replication of PB2-vMSL vRNA. Images of representative Cells A5, A6, A8, and A9 in Fig. 4 and Supplementary Fig. 4, respectively taken after IAV infection at Hours 7 and 10 (Cell A5); Hours 3 and 6 (Cell A6); Hours 9 and 11 (Cell A8), and Hours 5 and 7 (Cell A9). Images are enlarged to show RD punctate signals and are displayed as the square of maximum intensity projections across the z-planes in ImageJ. Scale bar: 10 µm.

Supplementary Figure 7. Time-lapse analysis of PB-vMSL vRNA replication in live cells after IAV infection. A549(MCP-GFPdN) cells were transfected with pHH-PB2-vMSL and infected with 2 MOI of PR8 IAV at 20 h post-transfection. At 2 h after infection, cells were imaged every hour for 18 h using a Nikon TiE eclipse inverted microscope. RDs (replicated dots) and ADs (apoptosis dots) are indicated by arrows. ADs are primarily static, and are larger in size and have higher signal intensities than RDs. The images of representative Cells A8 (A), A9 (B), and A10 (C) were selected, and are shown as the square of maximum intensity projections across the z-planes in ImageJ. Scale bar: 10 µm.

Supplementary Figure 8. Replication of PB2-vMSL vRNA after IAV infection. Intensity and number of punctate signals RDs in each z-plane of representative Cells A8 (A), A9 (B), and A10 (C) during the entire 18-h imaging period were measured using the Computer-Assisted Plasmid Summation (CAPS) program, and then counted, summed up, and graphed. Triangles indicate when apoptosis was first observed, and arrowheads indicate when vRNA replication was observed to begin.

Supplementary Figure 9. Expression of viral mRNA in non-apoptotic and apoptotic cells after IAV infection. A549 cells were infected with PR8 at an MOI of 2 (A, B) or treated with 800 nM of staurosporine (STP) (C) for 2 hours. Apoptotic (A, C) and non-apoptotic cells (B) were sorted by flow cytometry after Annexin V staining. After isolation of total RNA from the cells, mRNA was enriched by Oligo dT and analyzed with RNA-seq analysis (MGIseq).

Supplementary Figure S1

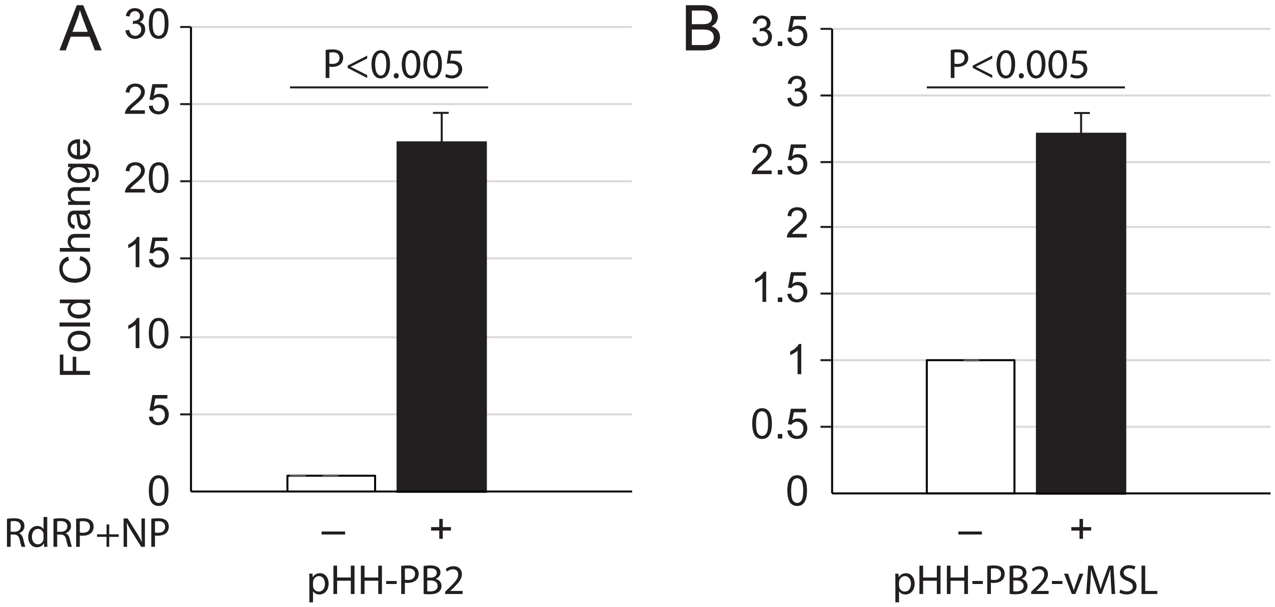

Supplementary Figure S2

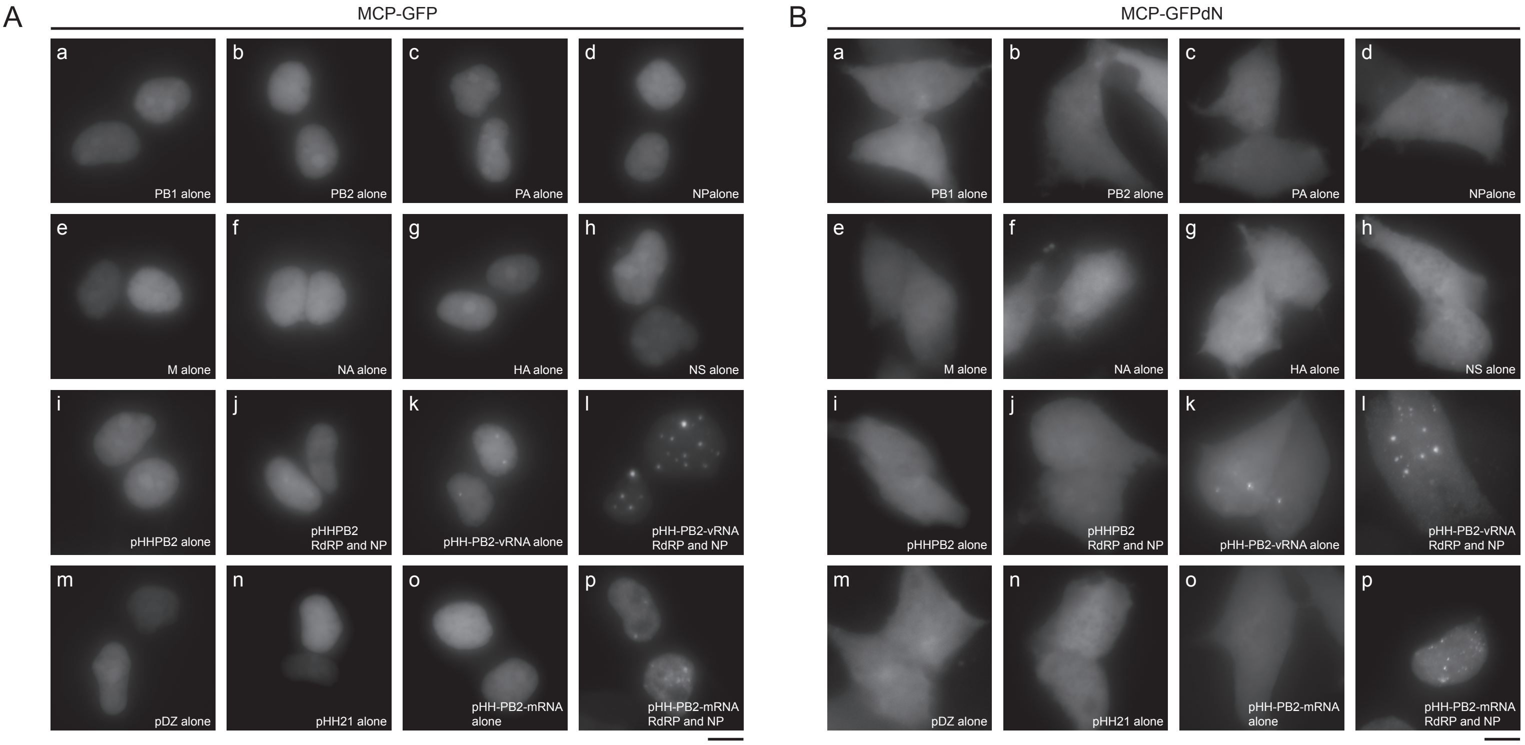

Supplementary Figure S3

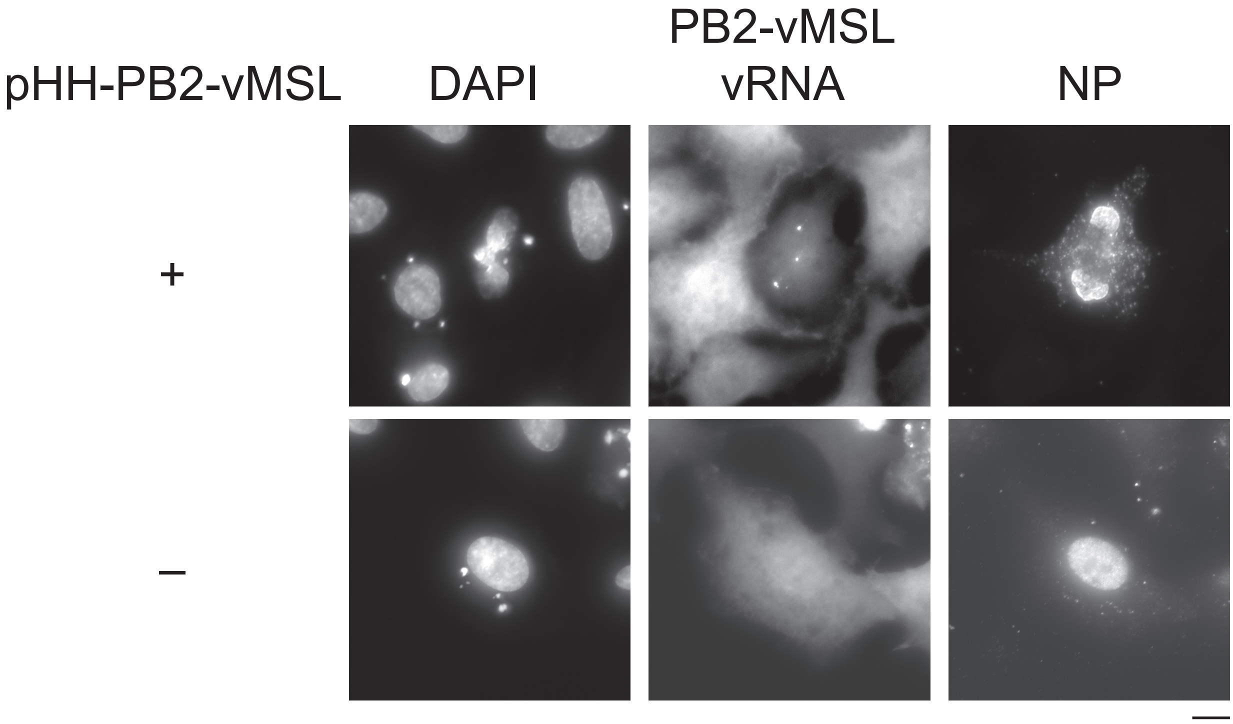

Supplementary Figure S4

Cell A2 5 h

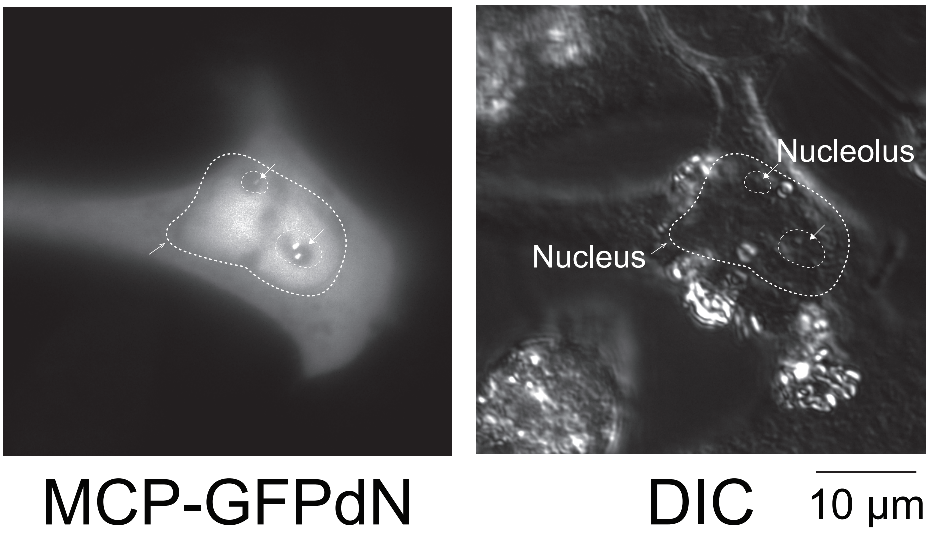

Supplementary Figure S5

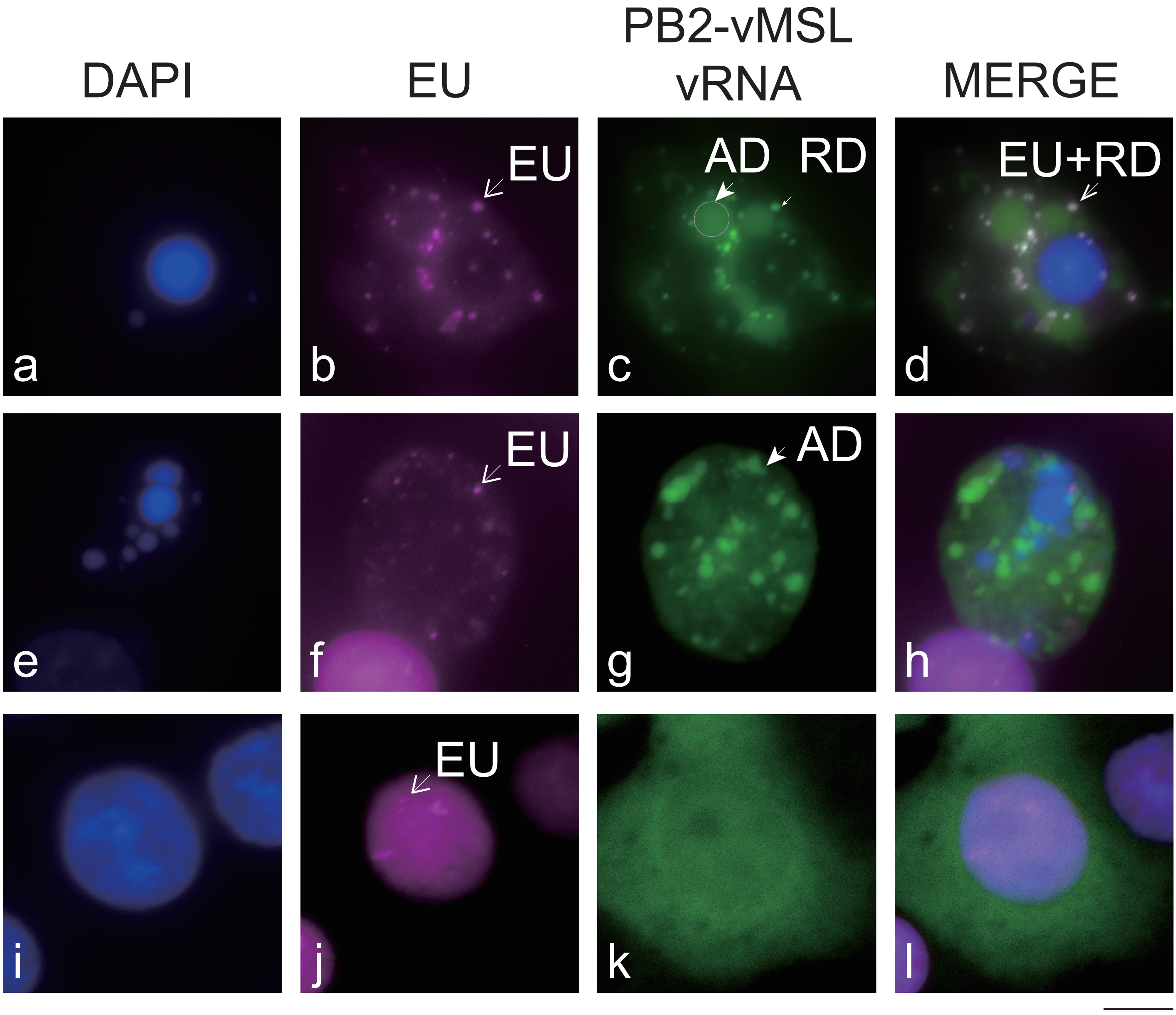

Supplementary Figure S6

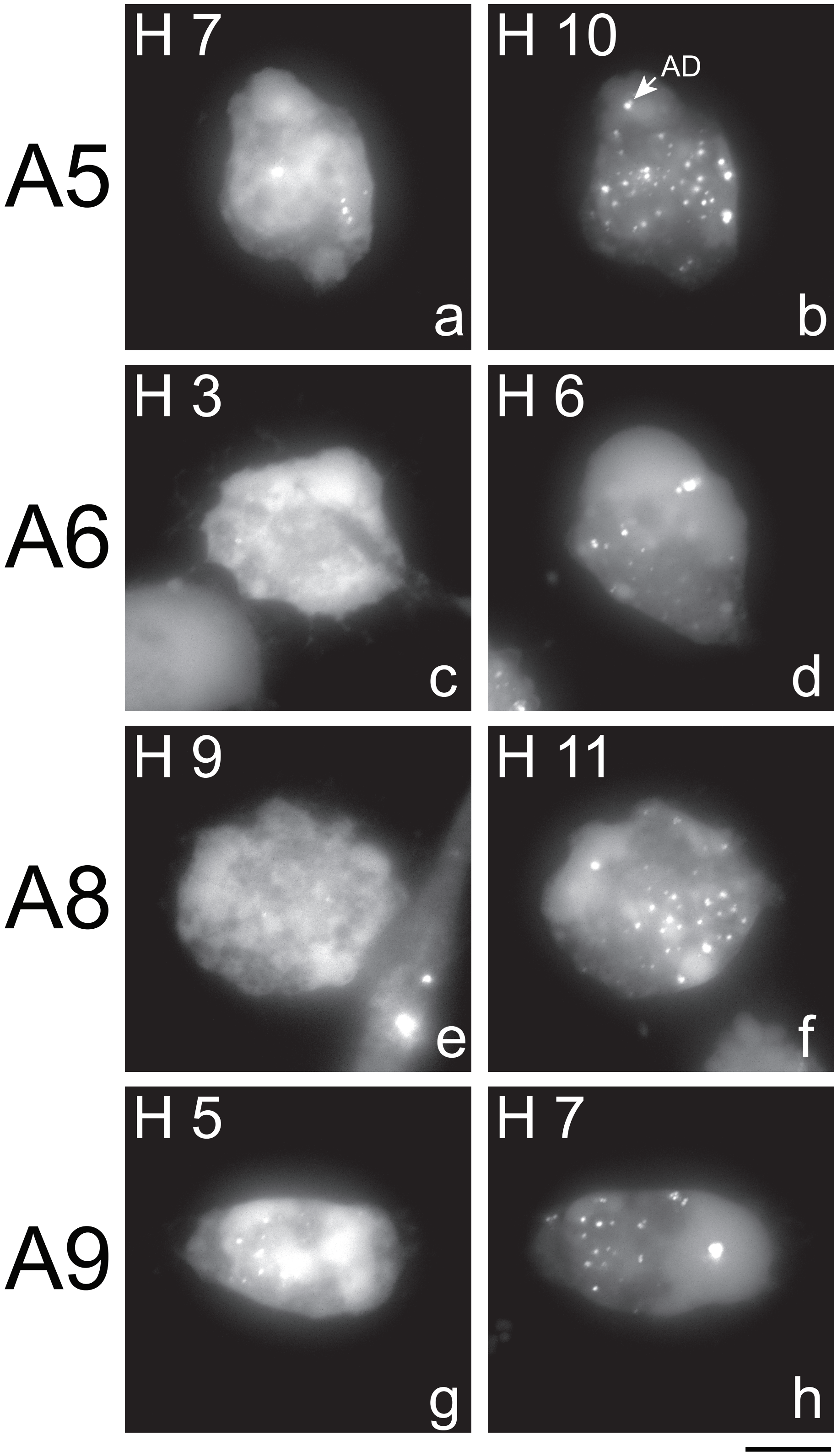

Supplementary Figure S7

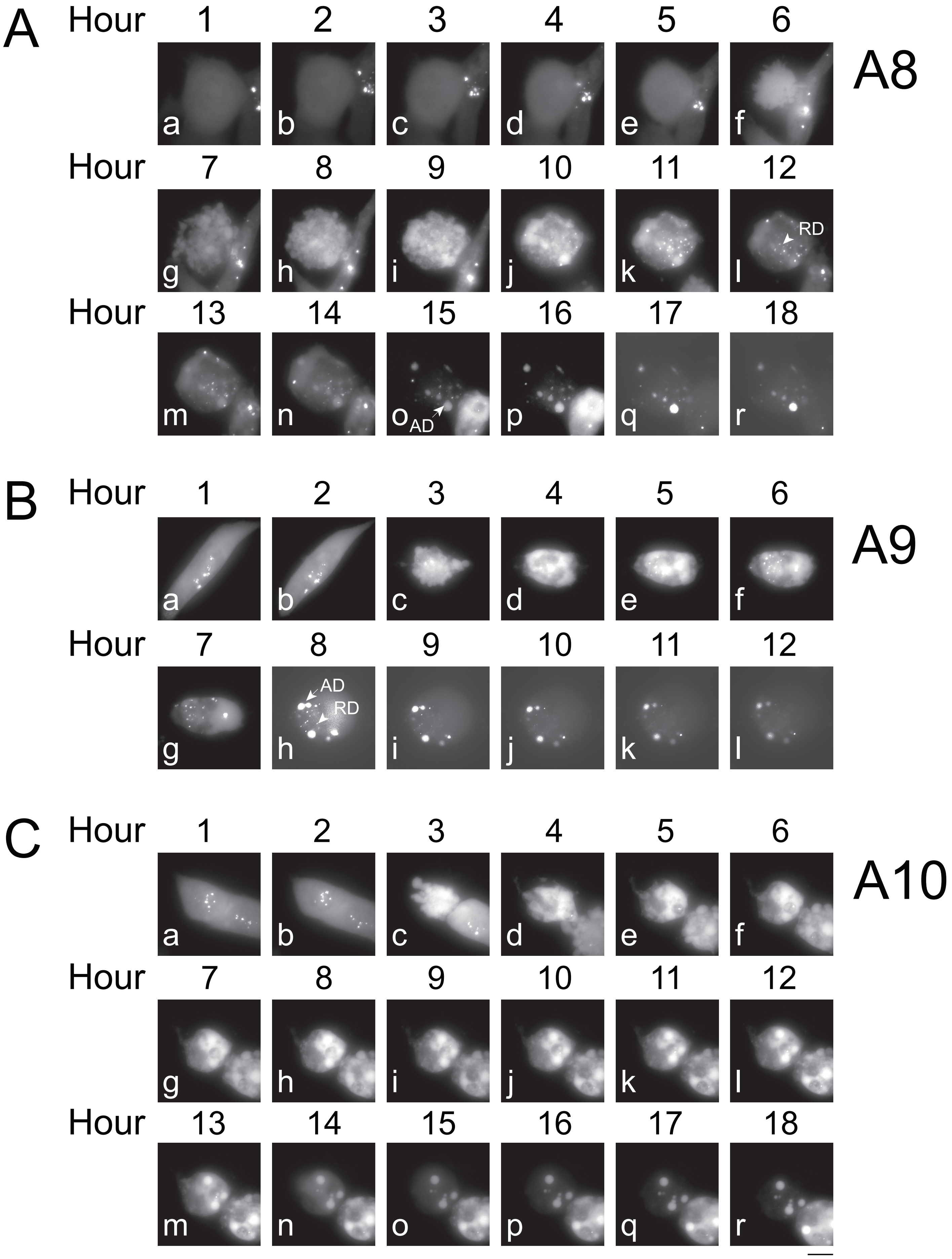

Supplementary Figure S8

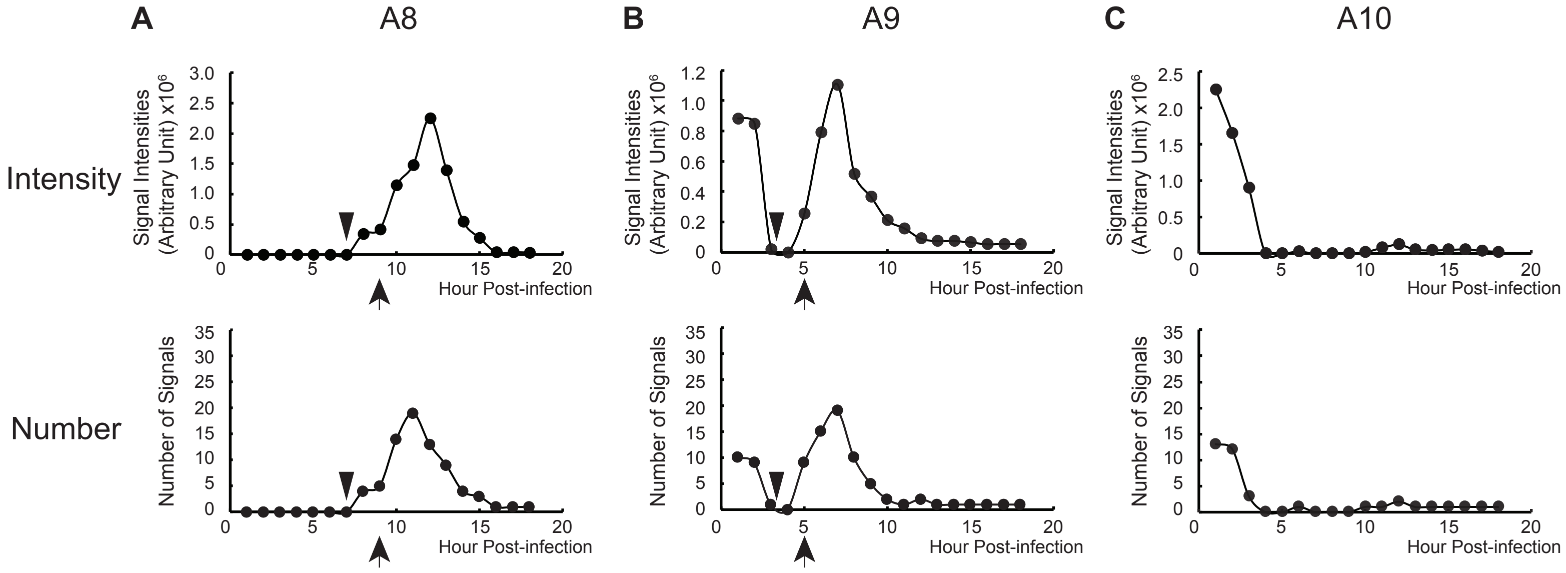

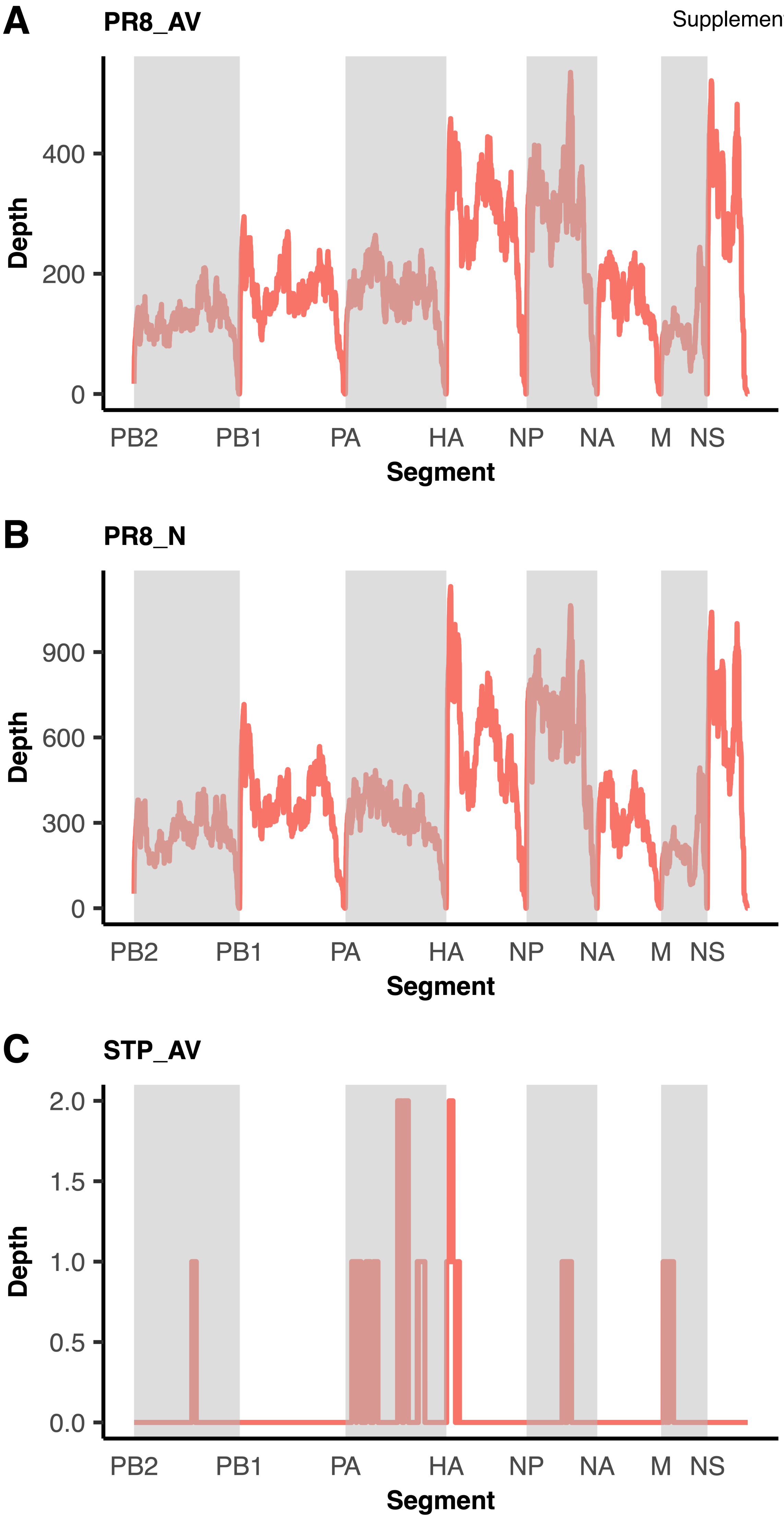
A
PR8_AV
Depth
400
200
0
PB2
PB1
PA
HA
NP
NA
M
NS
Segment
B
PR8_N
Depth
900
600
300
0
PB2
PB1
PA
HA
NP
NA
M
NS
Segment
C
STP_AV
Depth
2.0
1.5
1.0
0.5
0.0
PB2
PB1
PA
HA
NP
NA
M
NS
Segment

Supplementary Table 1. Distribution of PB2-vMSL vRNA signals

	PB2-vMSL vRNA signals in the cytoplasm					
Cotransfection[a]	–		RdRp and NP plasmids		All IAV Plasmids	
	Cells with Signals	Signals in Cytoplasm	Cells with Signals	Signals in Cytoplasm	Cells with Signals	Signals in Cytoplasm
**HEK293T**						
MCP-GFP						
Experiment 1	122/313 (39%)	0/122 (0%)	215/304 (71%)	0/215 (0%)	239/303 (79%)	0/239 (0%)
Experiment 2	144/306 (48%)	0/146 (0%)	219/309 (71%)	0/219 (0%)	210/309 (68%)	0/210 (0%)
Experiment 3	138/306 (45%)	0/138 (0%)	200/307 (65%)	0/200 (0%)	176/320 (55%)	0/176 (0%)
MCP-GFPdN						
Experiment 1	97/318 (31%)	0/97 (0%)	111/295 (38%)	0/111 (0%)	103/314 (33%)	5/103 (5%)
Experiment 2	126/274 (46%)	0/126 (0%)	109/271 (40%)	0/109 (0%)	154/306 (50%)	15/154 (10%)
Experiment 3	156/306 (51%)	0/156 (0%)	146/304 (48%)	0/146 (0%)	146/304 (48%)	9/146 (6%)
**A549**						
MCP-GFPdN						
Experiment 1	52/68 (76%)	0/52 (0%)	54/72 (75%)	0/54 (0%)	55/89 (62%)	8/55 (15%)
Experiment 2	53/81 (65%)	0/53 (0%)	54/81 (67%)	0/54 (0%)	58/88 (66%)	4/58 (7%)
Experiment 3	57/71 (80%)	0/57 (0%)	55/67 (82%)	0/55 (0%)	56/73 (77%)	10/56 (18%)

[a] Cells were cotransfected with pHH-PB2-vMSL and either pMCP-GFP or pGFP-MCPdN.
[b] *p*-value was determined by Student's *t*-test; *p* values for signals in cytoplasm in cells cotransfected with RdRp/NP plasmids and all plasmids for HEK293T cells (MCP-GFPdN) was 0.044; A549, 0.056.

Supplementary Table 2. Intensities of PB2-vMSL signals in A59(MCP-GFPdN) cells infected by PR8 IAV

Time[a]	Number of signals[b]	Sum of intensities[b]	Intensities of individual signal in A5 cell [Arbitrary Unit (AU)][c]
Hour 1	3	103241	29231; 26338; 47672
Hour 2	2	76537	57150; 19387
Hour 3	2	184961	74385; 110576
Hour 4	1	59411	59411
Hour 5	3	193538	152723; 19973; 20842
Hour 6	2	221393	133138; 88255
Hour 7	4	353578	20096; 82381; 43901; 207200
Hour 8	12	744094	74503; 110123; 98694; 43443; 35734; 16065; 162225; 68899; 17661; 49550; 39853; 27344
Hour 9	27	2669471	405688; 152052; 157191; 66543; 49664; 119170; 39167; 68092; 37632; 69427; 84062; 53479; 31336; 20091; 199137; 211714; 185500; 149899; 48876; 49616; 56314; 146927; 80912; 49786; 33382; 68719; 35095
Hour 10	37	4352624	519176; 87302; 244955; 56764; 14667; 22240; 209967; 98484; 86797; 30344; 138789; 82698; 23709; 28692; 51243; 35708; 164234; 157326; 108654; 305665; 161764; 130836; 118204; 226374; 73052; 101925; 112707; 183057; 80463; 124470; 168424; 87587; 71993; 52516; 68828; 46558; 76452
Hour 11	21	5145448	393475; 92277; 69174; 23892; 39017; 276339; 74130; 61944; 29002; 61039; 49082; 324695; 71952; 196021; 183419; 42031; 111240; 1288880; 314384; 439442; 1004013
Hour 12	19	1503479	50831; 37420; 11641; 16616; 18944; 19377; 42087; 160628; 38323; 14240; 25758; 25070; 108253; 93169; 58216; 441152; 143891; 102895; 94968
Hour 13	11	864551	29073; 15424; 77897; 28278; 21989; 85571; 60792; 41126; 188037; 249346; 67018
Hour 14	11	746903	23447; 17057; 126573; 59900; 12307; 62212; 33556; 24851; 125371; 236630; 24999
Hour 15	8	428182	25975; 98972; 53160; 36494; 24294; 24333; 130180; 34774
Hour 16	7	394791	14956; 73415; 43277; 30423; 20502; 168305; 43913
Hour 17	6	307674	52001; 35768; 25608; 82637; 90259; 21401
Hour 18	5	219663	33868; 29490; 21257; 66993; 68055

[a,] A549(MCP-GFPdN) cells were transfected with pHH-PB2-vMSL for 20 hours and followed every hour for 18 hours after PR8 IAV infection.
[b,] Total number and intensity of signals containing replicated dots (RDs) in cells were determined by CAPS.
[c,] Arbitrary Unit (A.U.) of signals were determined by CAPS.

Supplementary Table 3. Presence of PB2-vMSL vRNA in IAV.

	IAV(PB2)[a]		IAV(PB2-vMSL)	
Virion[c]	P1[b]	P2	P1	P2
PB2 segment[d]	28	205	66	246

[a] HEK293T cells were cotransfected with pQCXIH-PB2 and 7 pDZ reverse-genetic plasmids without pDZ-PB2, which was substituted with pHH21 (an empty vector), pHH-PB2, or pHH-PB2-vMSL. At 3 days post-transfection, IAV(null), IAV(PB2), and IAV(PB2-vMSL) viral particles were respectively collected.

[b] Viruses harvested from HEK293T cells were used to infect MDCK(PB2) cells. Viruses were collected at 3 days after infection (P1), and were amplified again in MDCK(PB2) cells to obtain viruses in P2.

[c] vRNA in virions was isolated and quantified by RT-qPCR. The $2^{-\Delta Ct}$ values were calculated relative to IAV(null) to determine the fold differences.

[d] Values indicated are fold differences compared with virions from cells infected by IAV(null) viruses.
